# Data-driven material modeling based on the Constitutive Relation Error

**DOI:** 10.1186/s40323-024-00279-x

**Published:** 2024-12-18

**Authors:** Pierre Ladevèze, Ludovic Chamoin

**Affiliations:** 1 CentraleSupélec, ENS Paris-Saclay, CNRS, LMPS-Laboratoire de Mécanique Paris-Saclay, Université Paris-Saclay, 4 Avenue des Sciences, 91190 Gif-sur-Yvette, France; 2 Institut Universitaire de France (IUF), 1 rue Descartes, 75231 Paris, France

**Keywords:** Data-driven modeling, Materials science, Constitutive Relation Error, Elasto-(visco-)plasticity

## Abstract

Prior to any numerical development, the paper objective is to answer first to a fundamental question: what is the mathematical form of the most general data-driven constitutive model for stable materials, taking maximum account of knowledge from physics and materials science? Here we restrict ourselves to elasto-(visco-)plastic materials under the small displacement assumption. The experimental data consists of full-field measurements from a family of tested mechanical structures. In this framework, a general data-driven approach is proposed to learn the constitutive model (in terms of thermodynamic potentials) from data. A key element that defines the proposed data-driven approach is a tool: the Constitutive Relation Error (CRE); the data-driven model is then the minimizer of the CRE. A notable aspect of this procedure is that it leads to quasi-explicit formulations of the optimal constitutive model. Eventually, a modified Constitutive Relation Error is introduced to take measurement noise into account.

## Introduction

A first pillar in computational mechanics is a certain abstract representation and understanding of the world we live in, in terms of models described by physics laws but also sometimes debatable theories and knowledge developed over the centuries. In this framework, the equilibrium equations, the compatibility equations and the two principles of the thermodynamics of irreversible processes constitute a foundation on which, in our opinion, any approach should be based. A second pillar is experimental data and its impact is continuously increasing nowadays. Data can be of different natures; indeed, it may refer to the geometry of the studied structure, its loading, or the response of the materials constituting the structure. Any calculation model is situated between these two pillars with more or less weight for one or the other. When the influence of data is largely dominant, and no questionable hypothesis is added in the modeling, we generally speak of data-driven approaches. This way, the use of parameterized behavior models from materials science does not seem to us to be a data-driven approach.

Here, we precisely focus on the material. We consider the data-driven modeling of stable complex materials which has become a growing trend. The pioneer works were due to M. Ortiz and F. Chinesta and their co-workers [39, 41, 42, 53, 57]. In these works, materials science was either discarded or understated. Further works following this line introduced a structuration of data [15]. Alternative developments have recently emerged, in which some physics background is involved when learning the constitutive model. Pioneer works in this direction are [26, 40] and the research topic is evolving fast with various techniques coupling deep learning and physics information to describe the behavior of materials using neural networks e.g., [8, 28, 38, 44, 55, 68, 76, 78].

Looking deeper in the bibliography of “physics-augmented” learning, in which physics knowledge (e.g., principles of thermodynamics or symmetries) is integrated in the neural network architecture, a large list of works is available e.g., [9, 31, 32, 54] to cite some very recent ones. We may separate contributions depending on the data used. A first set of contributions aims to train neural networks in a supervised learning procedure with a strain–stress (or strain-free energy) database generated from a known constitutive model [3, 23, 60, 76]; nevertheless, getting such measurement couples under complex loading is hardly possible in practice. Another set of contributions addresses unsupervised training of neural networks for constitutive modeling [8, 38, 75]; for instance, in the EUCLID approach proposed in [21, 75], full-field displacement observations are processed to derive strain and stress fields employed as the learning input with a loss function that penalizes the non-satisfaction of equilibrium.

In contrast to the previous pioneer approaches, the aim of this paper is to define a data-driven mathematical model prior to any numerical development, taking maximum account of knowledge on physics and materials science. This paper is thus limited to some fundamental aspects; numerical treatment, which involves solving optimization, regression and interpolation problems for which machine learning tools (and in particular deep neural networks) complement the more conventional tools, is not covered. Here we restrict ourselves to stable elasto-(vico)plastic materials under the small displacement assumption. The central concept of the proposed approach is a tool, referred to as the Constitutive Relation Error (CRE), the computed data-driven constitutive model being the minimizer of the CRE.

The CRE has been initially introduced in model verification for linear problems [45]. Precisely, the CRE is built on the separation of the governing equations of the mechanical or physical problem, defined over the space-time domain: equilibrium and compatibility equations on the one hand, and constitutive equations on the other hand. In contrast to the constitutive relations which are empirical and strongly depend on experimental data, the other equations can be qualified as “exact”. Suitable approximations should thus be constructed that “exactly” satisfy the “exact equations”; their quality can be assessed by measuring the mismatch in constitutive equations by the CRE tool. Among the numerous works on CRE for model verification, one can mention the reviews available in the book [48], and the chapters [12, 49]. Direct applications of the CRE for model updating in elasticity and elastodynamics have been performed in the 1990s, in the context of parametrized constitutive relations; one can cite [6, 10, 22, 25, 43]. An extension of the CRE to unreliable data by using a modified formulation (mCRE) has been introduced in [13, 46] for the updating of vibration models. Since then, numerous further works have been performed over the years for model updating and validation for linear or nonlinear materials, in quasi-statics but also in dynamics e.g., [2, 7, 11, 16, 17, 19, 37, 59, 66, 72] to cite a few of them. Recent applications to sequential data assimilation and structural health monitoring by complementing mCRE with Kalman filtering have also been investigated lately [18, 58].

Like all inverse problems, data-driven material modeling is characterized by an overabundance of data and therefore leads to the solution of ill-posed problems. CRE was primarily used to provide a solution to such problems (in fact to the usual family of ill-posed problems called standard), in situations where the constitutive relation was known; the goal was then to identify or update the associated parameters. The experimental data derived from full-field measurements (even though sparser measurements can also be considered) is related to the family of structures tested, made from the same material and subjected to different loads. Here, we show that the CRE framework can again be used to calculate the data-driven model that is defined for stable materials by thermodynamic potentials, the calculated potentials being the CRE minimizer. The advantage of the approach is the use of an error metric which is directly focused on what needs to be learnt, that is the constitutive relation. To start, we consider linear and nonlinear elastic materials with convex energy, for which the CRE approach gives original solutions. This approach is compared with that proposed in the pioneer work [41].

For elasto-(visco-)plastic materials, materials science is a great help. The very common internal variables framework may be advantageously used but data-driven approaches involve two main difficulties. First, the hidden internal state variables are not known a priori, nor is their number. Moreover, they are not intrinsic, and transformations on these variables leading to the same material model are possible. This is why, for stable materials with a convex energy, we introduce the so-called normal formulation of the hidden internal variables resulting from a transformation of these variables while satisfying, of course, the two principles of the thermodynamics of irreversible processes. In addition, we follow what is classically done in materials science to describe stable elasto-(visco-)plastic materials by referring to the sub-family of standard materials introduced in [29]. It follows that the mathematical shape of data-driven models for stable elasto-(visco-)plastic materials is defined by only one convex function. This formulation, both normal and standard, is at the core of the proposed data-driven CRE approach. A unique solution is given to the standard ill-posed problems, as the minimizer of the CRE functional; it is defined by its own constitutive relation, which differs from the reference relation. The optimal data-driven model is then computed by a constrained minimization of the CRE using convex optimization.

Eventually, we consider noisy data. Following [33, 71], we assume that the main source of noise comes from full-field measurements. A modified Constitutive Relation Error (mCRE) is then introduced, still for elasto-(visco-)plastic materials, and developments are very similar to the case of noiseless data for the derivation of a data-driven constitutive model.

To close this introduction, let us mention that some first numerical investigations from the CRE-based strategy have been conducted for particular situations in [8, 9], using input convex neural networks (ICNNs) to represent the learnt thermodynamic potentials (so-called NN-mCRE strategy). We also mention that the generalized standard material framework which is considered in this work is closely related to the (more general) GENERIC thermodynamic framework introduced in [27, 67, 70] and developed in solid mechanics in [63]; it was used in several works for data-driven modeling e.g., [14, 32].

## Preliminaries: standard ill-posed problems and CRE concept

Material identification requires to solve problems with over-abundant data i.e., ill-posed computational problems. It is well-known that the classical formulation of such problems, without adding any regularization process, does not admit a solution. We will see that the Constitutive Relation Error (CRE) is able to give a solution, the constitutive relation being relaxed.

### Notations

The tested structure we shall study occupies the domain $\Omega \subset \textbf{R}^3$ with boundary $\partial \Omega$, a typical point in $\Omega$ being denoted $\underline{M}$. The vectors, for instance displacements or velocities, are denoted by $\underline{U}$, $\underline{V}$, etc. Linear operators or second-order tensors, for instance strain or stress tensors, are written $\epsilon \hspace{-2.5pt}{\epsilon }$, $\sigma \hspace{-5.0pt}{\sigma }$, etc. More complex tensors or operators are written in bold text, such as the Hooke tensor which is written $\textbf{K}$. This previous notation is also used for the different sets or spaces e.g., $\textbf{R}^3$.

The Euclidean transpose is denoted by the superscript $^T$, so that the scalar product between vectors $\underline{V}$ and $\underline{W}$ reads $\underline{V}^T\underline{W}$. If there is no ambiguity, this scalar product can also be written $\underline{V}\cdot \underline{W}$. Work and power bring into play the trace operator such that:

1 $$\begin{aligned} \text {Tr}[\sigma \hspace{-5.0pt}{\sigma }\epsilon \hspace{-2.5pt}{\epsilon }] = \sum _{i,j\in \{1,2,3\}}[\sigma \hspace{-5.0pt}{\sigma }]_{ij}[\epsilon \hspace{-2.5pt}{\epsilon }]_{ij} \end{aligned}$$

In this paper, we shall consider small perturbations which means that the different configurations occupied by the structure can be replaced by the initial configuration $\Omega$. To fix ideas, the tested structure is placed in an environment characterized by:

- a displacement $\underline{U}_d$ on the part $\partial _1 \Omega$ of the boundary $\partial \Omega$ ($\underline{U}_d \in [\textbf{H}^{1/2}(\partial _1 \Omega )]^3$);
- a surface force $\underline{F}_d$ on the part $\partial _2 \Omega$ of the boundary $\partial \Omega$ ($\underline{F}_d \in [\textbf{H}^{-1/2}(\partial _2 \Omega )]^3$);
- a body force $\underline{f}_d$ over $\Omega$ (here we take $\underline{f}_d=\underline{0}$ for the sake of simplicity).

### Standard ill-posed problems

Commonly in material identification, the displacement field $\underline{U}_d$ is obtained from full-field measurements for which very efficient techniques are available today (see [33, 71]). Moreover, boundary data is overabundant such that:

2 $$\begin{aligned} \partial _1 \Omega \cup \partial _2 \Omega = \partial \Omega ; \quad \partial _1 \Omega \cap \partial _2 \Omega \equiv \partial _{12} \Omega \ne \emptyset \end{aligned}$$

which characterizes a standard ill-posed problem. These conditions are not mandatory for the use of the CRE concept, but they give certain properties that can help the calculation process. Of course, they can be extended to mixed boundary conditions. Incomplete conditions can also be introduced, but for this last case, the computational problem is no more standard.

In general, the part of the boundary where displacement full-field measurement takes place is also free, and therefore belongs to the part $\partial _{12} \Omega$ of the boundary where force is also known. A very common situation in identification problems is that the force is known over the entire boundary, eventually free on some parts, and the displacement field is measured on a part of the boundary. If the intersection of the parts of the boundary where the displacement is prescribed ($\partial _1 \Omega$) and the force is given ($\partial _2 \Omega$) is empty, i.e. $\partial _{12} \Omega = \emptyset$, the computational problem becomes well-posed if the boundary conditions cover the entire boundary; it has a solution for classical material models.

#### Remark 1

We emphasize that a well-posed problem in elasticity, for example, is a problem for which the boundary conditions are such that it exists a unique solution for any constitutive relation. In other words, the knowledge of the boundary conditions does not give any information about the constitutive relation. This explains why one uses, in material identification, boundary conditions leading to an ill-posed computational problem.

Let us introduce $\varvec{\mathcal {U}}_{ad}^K$ the space of kinematically admissible displacement fields:

3 $$\begin{aligned} \varvec{\mathcal {U}}_{ad}^K \equiv \left\{ \underline{U}\;\text {s.t.}\; \underline{U}\in [\textbf{H}^1(\Omega )]^3, \underline{U}_{|\partial _1 \Omega } = \underline{U}_d \right\} \end{aligned}$$

This affine space can be written:

4 $$\begin{aligned} \varvec{\mathcal {U}}_{ad}^K = \underline{U}_0^K + \varvec{\mathcal {U}}_{ad,0}^K \end{aligned}$$

where $\underline{U}_0^K \in \varvec{\mathcal {U}}_{ad}^K$ is a particular admissible displacement field, and $\varvec{\mathcal {U}}_{ad,0}^K$ is the associated vectorial space.

Now, let us consider the space of statically admissible stress fields:

5 $$\begin{aligned} \varvec{\mathcal {S}}_{ad}^S \equiv \left\{ \sigma \hspace{-5.0pt}{\sigma }\;\text {s.t.}\; \sigma \hspace{-5.0pt}{\sigma }\in [\textbf{H}(\text {div\,},\Omega )]^3, \underline{\text {div\,}} \sigma \hspace{-5.0pt}{\sigma }= \underline{0} \; \text {over}\, \Omega , \sigma \hspace{-5.0pt}{\sigma }\underline{n}_{|\partial _2 \Omega } = \underline{F}_d \right\} \end{aligned}$$

which can be defined from the principle of virtual works:

6 $$\begin{aligned} { \int _{\Omega }\text {Tr}[\sigma \hspace{-5.0pt}{\sigma }\epsilon \hspace{-2.5pt}{\epsilon }(\underline{U})] \text {d}\Omega = \int _{\partial _2 \Omega } \underline{F}_d \cdot \underline{U}\text {d}S\quad \forall \underline{U}\in \varvec{\mathcal {U}}_{ad,0}^S } \end{aligned}$$

thanks to the virtual displacement space:

7 $$\begin{aligned} \varvec{\mathcal {U}}_{ad,0}^S \equiv \left\{ \underline{U}\;\text {s.t.}\; \underline{U}\in [\textbf{H}^1(\Omega )]^3, \underline{U}_{|\partial _1 \Omega \backslash \partial _{12} \Omega } = \underline{0} \right\} \end{aligned}$$

It follows the fundamental property of standard ill-posed problems:

#### Property 1

For standard ill-posed problems, one has:

8 $$\begin{aligned} \varvec{\mathcal {U}}_{ad,0}^K \subset \varvec{\mathcal {U}}_{ad,0}^S \end{aligned}$$

### The Constitutive Relation Error

Let us consider the simple situation of elastic materials which may be linear or nonlinear; one has:

9 $$\begin{aligned} \sigma \hspace{-5.0pt}{\sigma }= \textbf{K}(\epsilon \hspace{-2.5pt}{\epsilon }) \; \text {over}\, \Omega \end{aligned}$$

where the Hooke operator $\textbf{K}$ is supposed to be known for the moment. An alternative formulation following J.J. Moreau’s work [64] is to describe the elastic material behavior thanks to two potentials $\Psi (\epsilon \hspace{-2.5pt}{\epsilon })$ and $\Psi ^*(\sigma \hspace{-5.0pt}{\sigma })$ which are dual convex functions. This duality is defined by the work bilinear form:

10 $$\begin{aligned} (\epsilon \hspace{-2.5pt}{\epsilon },\sigma \hspace{-5.0pt}{\sigma }) \in \textbf{E}\times \textbf{F}\longmapsto \int _{\Omega }\text {Tr}[\sigma \hspace{-5.0pt}{\sigma }\epsilon \hspace{-2.5pt}{\epsilon }] \text {d}\Omega \in \textbf{R}\end{aligned}$$

One has the following well-known property:

#### Property 2

Let $\Psi$ and $\Psi ^*$ be two dual convex functions related to the work bilinear form. The CRE functional is then defined as:

11 $$\begin{aligned} E^2_{CRE}(\underline{U},\sigma \hspace{-5.0pt}{\sigma }) \equiv \Psi (\epsilon \hspace{-2.5pt}{\epsilon }(\underline{U})) + \Psi ^*(\sigma \hspace{-5.0pt}{\sigma }) - \int _{\Omega }\text {Tr}[\sigma \hspace{-5.0pt}{\sigma }\epsilon \hspace{-2.5pt}{\epsilon }(\underline{U})]\text {d}\Omega \ge 0 \quad \forall (\epsilon \hspace{-2.5pt}{\epsilon },\sigma \hspace{-5.0pt}{\sigma }) \in \textbf{E}\times \textbf{F}\end{aligned}$$

Moreover, (i) and (ii) are equivalent:

12 $$\begin{aligned} \begin{aligned}&(i)\; \sigma \hspace{-5.0pt}{\sigma }= \textbf{K}(\epsilon \hspace{-2.5pt}{\epsilon }) \; \text {over}\; \Omega \\&(ii)\; E_{CRE}(\underline{\nu },\sigma \hspace{-5.0pt}{\sigma }) = 0 \end{aligned} \end{aligned}$$

$E_{CRE}$ is the global Constitutive Relation Error.

The classical formulation of the computational problem does not admit a solution for ill-posed problems.The main interest of the CRE method is to give a solution to such ill-posed problems by relaxing the constitutive relation. This is defined by:

13 $$\begin{aligned} \boxed { (\underline{U}^K,\sigma \hspace{-5.0pt}{\sigma }^S) \in \varvec{\mathcal {U}}^K_{ad} \times \varvec{\mathcal {S}}^S_{ad} \equiv \mathop {\mathrm {arg\,min}}\limits _{(\tilde{\underline{U}}^K,\tilde{\sigma \hspace{-5.0pt}{\sigma }}^S) \in \varvec{\mathcal {U}}^K_{ad} \times \varvec{\mathcal {S}}^S_{ad}}E^2_{CRE}(\tilde{\underline{U}}^K,\tilde{\sigma \hspace{-5.0pt}{\sigma }}^S) } \end{aligned}$$

The value of the minimum $E^2_{CRE}(\underline{U}^K,\sigma \hspace{-5.0pt}{\sigma }^S)$ is not equal to 0 in general for ill-posed computational problems.

Let us note that in the specific case of a linear elastic material behavior with $\sigma \hspace{-5.0pt}{\sigma }= \textbf{K}\epsilon \hspace{-2.5pt}{\epsilon }$, $\textbf{K}$ being the Hooke elasticity tensor, corresponding potentials read $\Psi (\epsilon \hspace{-2.5pt}{\epsilon }) = \frac{1}{2}\int _{\Omega }\text {Tr}[\textbf{K}\epsilon \hspace{-2.5pt}{\epsilon }\epsilon \hspace{-2.5pt}{\epsilon }]\text {d}\Omega$ and $\Psi ^*(\sigma \hspace{-5.0pt}{\sigma }) = \frac{1}{2}\int _{\Omega }\text {Tr}[\textbf{K}^{-1} \sigma \hspace{-5.0pt}{\sigma }\sigma \hspace{-5.0pt}{\sigma }] \text {d}\Omega$, which yields:

14 $$\begin{aligned} E^2_{CRE}(\underline{U},\sigma \hspace{-5.0pt}{\sigma }) = \frac{1}{2} \int _{\Omega }\text {Tr}[(\sigma \hspace{-5.0pt}{\sigma }- \textbf{K}\epsilon \hspace{-2.5pt}{\epsilon }(\underline{U})) \textbf{K}^{-1} (\sigma \hspace{-5.0pt}{\sigma }- \textbf{K}\epsilon \hspace{-2.5pt}{\epsilon }(\underline{U}))] \text {d}\Omega \end{aligned}$$

### Computation of the optimal solution $(\underline{U}^K,\sigma \hspace{-5.0pt}{\sigma }^S)$

In this section, we deal with how to practically compute the optimal solution of (13) for ill-posed computational problems.

From Property 1, we get the following decoupling property for any admissible pair $(\tilde{\underline{U}}^K,\tilde{\sigma \hspace{-5.0pt}{\sigma }}^S) \in \varvec{\mathcal {U}}^K_{ad} \times \varvec{\mathcal {S}}^S_{ad}$:

15 $$\begin{aligned} E^2_{CRE}(\tilde{\underline{U}}^K,\tilde{\sigma \hspace{-5.0pt}{\sigma }}^S) = J_1(\tilde{\underline{U}}^K) + J_2(\tilde{\sigma \hspace{-5.0pt}{\sigma }}^S) \end{aligned}$$

with:

16 $$\begin{aligned} {\begin{aligned} J_1(\tilde{\underline{U}}^K) \equiv \,&\frac{1}{2}\int _{\Omega }\text {Tr}[\epsilon \hspace{-2.5pt}{\epsilon }(\tilde{\underline{U}}^K) \textbf{K}\epsilon \hspace{-2.5pt}{\epsilon }(\tilde{\underline{U}}^K)] \text {d}\Omega - \int _{\partial \Omega \backslash \partial _1 \Omega } \underline{F}_d \cdot \tilde{\underline{U}}^K \text {d}S\\  &+ \frac{1}{2} \int _{\partial \Omega \backslash \partial _1 \Omega } \underline{F}_d \cdot \underline{U}^K_0 \text {d}S\\ J_2(\tilde{\sigma \hspace{-5.0pt}{\sigma }}^S) \equiv \,&\frac{1}{2}\int _{\Omega }\text {Tr}[\tilde{\sigma \hspace{-5.0pt}{\sigma }}^S \textbf{K}^{-1} \tilde{\sigma \hspace{-5.0pt}{\sigma }}^S] \text {d}\Omega - \int _{\Omega }\text {Tr}[\tilde{\sigma \hspace{-5.0pt}{\sigma }}^S \epsilon \hspace{-2.5pt}{\epsilon }(\underline{U}^K_0)] \text {d}\Omega \\  &+ \frac{1}{2} \int _{\partial \Omega \backslash \partial _1 \Omega } \underline{F}_d \cdot \underline{U}^K_0 \text {d}S\end{aligned}} \end{aligned}$$

Indeed, for a given $\underline{U}^K_0 \in \varvec{\mathcal {U}}^K_{ad}$, we have $\tilde{\underline{U}}^K-\underline{U}^K_0 \in \varvec{\mathcal {U}}^K_{ad,0} \subset \varvec{\mathcal {U}}_{ad,0}^S$ which yields:

17 $$\begin{aligned} \int _{\Omega }\text {Tr}[\tilde{\sigma \hspace{-5.0pt}{\sigma }}^S \epsilon \hspace{-2.5pt}{\epsilon }(\tilde{\underline{U}}^K)] \text {d}\Omega = \int _{\Omega }\text {Tr}[\tilde{\sigma \hspace{-5.0pt}{\sigma }}^S \epsilon \hspace{-2.5pt}{\epsilon }(\underline{U}^K_0)] \text {d}\Omega + \int _{\partial \Omega \backslash \partial _1 \Omega } \underline{F}_d \cdot (\tilde{\underline{U}}^K-\underline{U}^K_0)\text {d}S\end{aligned}$$

The decoupling property (15) is classical for well-posed computational problems (defining potential and complementary energies), but we just showed that it is also valid for standard ill-posed problems. It yields that the minimization (13) results in the solution of two independent minimization problems. Moreover, instead of solving the $J_2$-problem, we can compute its displacement formulation obtained thanks to a new dualization; defining

18 $$\begin{aligned} J_1^S(\tilde{\underline{U}}^S) = \Psi (\epsilon \hspace{-2.5pt}{\epsilon }(\tilde{\underline{U}}^S)) - \int _{\partial _2\Omega } \underline{F}_d \cdot \tilde{\underline{U}}^S \text {d}\Omega \end{aligned}$$

and denoting by $\underline{U}^S$ the minimizer of $J_1^S$ over $\varvec{\mathcal {U}}_{ad,0}^S$, we get $\sigma \hspace{-5.0pt}{\sigma }^S = \textbf{K}(\epsilon \hspace{-2.5pt}{\epsilon }(\underline{U}^S))$.

Consequently, to compute the minimizer $(\underline{U}^K,\sigma \hspace{-5.0pt}{\sigma }^S)$ of the CRE in $\varvec{\mathcal {U}}^K_{ad} \times \varvec{\mathcal {S}}^S_{ad}$, one computes the displacement pair $(\underline{U}^K,\underline{U}^S)$ which is easy. Of course, the classical finite element method can be used to perform these minimizations.

## Data-driven material modeling—elastic materials

The CRE-method is now developed for stable elastic materials in order to learn (9) ($\textbf{K}$ is not given any more). First, we clearly specify the information which is available.

### Available information

#### Experimental data

We consider a series of tested structures $i \in \textbf{N}_{test}$ which occupy the same domain $\Omega$. For each tested structure, the experimental data is:

19 $$\begin{aligned} \underline{U}_d(i) \; \text {over}\, \partial _1 \Omega ; \quad \underline{F}_d(i) \; \text {over}\, \partial _2 \Omega \end{aligned}$$

#### Additional knowledge from materials science

The considered environment is such that the studied material can be supposed stable. It follows that the behavior of the material can be described by two dual convex functions $\Psi$ and $\Psi ^*$, the duality being defined by the work bilinear form. These two functions can be written:

20 $$\begin{aligned} \Psi (\epsilon \hspace{-2.5pt}{\epsilon }) = \int _{\Omega }\psi (\epsilon \hspace{-2.5pt}{\epsilon }) \text {d}\Omega ; \quad \Psi ^*(\sigma \hspace{-5.0pt}{\sigma }) = \int _{\Omega }\psi ^*(\sigma \hspace{-5.0pt}{\sigma }) \text {d}\Omega \end{aligned}$$

where $\psi$ and $\psi ^*$ are two dual convex functions related to the local work bilinear form. These define the elastic material behavior as:

21 $$\begin{aligned} \sigma \hspace{-5.0pt}{\sigma }= \frac{\partial \psi }{\partial \epsilon \hspace{-2.5pt}{\epsilon }} ; \quad \epsilon \hspace{-2.5pt}{\epsilon }= \frac{\partial \psi ^*}{\partial \sigma \hspace{-5.0pt}{\sigma }} \end{aligned}$$

Moreover, $\psi (\epsilon \hspace{-2.5pt}{\epsilon })$ is an energy and thus satisfies to:

22 $$\begin{aligned} \psi \ge 0 ; \quad \psi (0)=0 ; \quad {\frac{\partial \psi }{\partial \epsilon \hspace{-2.5pt}{\epsilon }}(0)=0} \end{aligned}$$

The last relation means that the stress $\sigma \hspace{-5.0pt}{\sigma }$ is zero when the strain $\epsilon \hspace{-2.5pt}{\epsilon }$ is also zero.

The corresponding admissible space for $\psi$ is denoted by $\varvec{\uppsi }$. It follows that the additional knowledge coming from materials science is reduced to:

23 $$\begin{aligned} \psi {\in }{\varvec{\uppsi }} \end{aligned}$$

where $\varvec{\uppsi }$ is a convex subset.

### Optimal data-driven material model

#### Definition of the computed data-driven model

The computed data-driven model is the minimizer of the CRE for the family of tested structures. First, one defines the following CRE for a given material model (described by the thermodynamic potential $\psi \in \uppsi$):

24 $$\begin{aligned} { \overline{E}^2_{CRE}(\psi ) \equiv \frac{1}{N_{test}} \sum _{i \in \textbf{N}_{test}} E^2_{CRE}\left( \psi ;\underline{U}^K(i),\sigma \hspace{-5.0pt}{\sigma }^S(i)\right) } \end{aligned}$$

with

25 $$\begin{aligned} { \left( \underline{U}^K(i),\sigma \hspace{-5.0pt}{\sigma }^S(i)\right) = \mathop {\mathrm {arg\,min}}\limits _{\left( \tilde{\underline{U}}^K(i),\tilde{\sigma \hspace{-5.0pt}{\sigma }}^S(i)\right) \in \varvec{\mathcal {U}}^K_{ad}(i) \times \varvec{\mathcal {S}}^S_{ad}(i)}E^2_{CRE}\left( \psi ;\tilde{\underline{U}}^K(i),\tilde{\sigma \hspace{-5.0pt}{\sigma }}^S(i)\right) } \end{aligned}$$

$\varvec{\mathcal {U}}^K_{ad}(i)$ and $\varvec{\mathcal {S}}^S_{ad}(i)$ being the admissible spaces related to problem $i \in \textbf{N}_{test}$.

The computed data-driven model is thus determined by:

26 $$\begin{aligned} { \boxed { \overline{\psi } \equiv \mathop {\mathrm {arg\,min}}\limits _{\psi \in \uppsi } \overline{E}^2_{CRE}(\psi ) }} \end{aligned}$$

The final value $\overline{E}^2_{CRE}(\overline{\psi })$ reached by the CRE (averaged over the tested structures) at the end of the optimization process characterizes the quality of the optimal data-driven material model. It is different from 0 in general; in the very special case where this error is zero, it means the computed material model is compatible with the experimental data.

The validation of the calculated data-driven model, although a fundamental question, is beyond the scope of this paper. Nevertheless, a first idea is to describe the validation domain $\overline{\textbf{s}}$ from the set $\textbf{s}_{exp}$ of strain–stress pairs associated to the solutions of the *i*-problems with $\overline{\psi } \in \uppsi$. One has:

27 $$\begin{aligned} { \overline{\textbf{s}} \equiv \left\{ (\epsilon \hspace{-2.5pt}{\epsilon },\sigma \hspace{-5.0pt}{\sigma }) \in \textbf{R}^6 \times \textbf{R}^6 | e_{CRE}(\overline{\psi };\epsilon \hspace{-2.5pt}{\epsilon },\tilde{\sigma \hspace{-5.0pt}{\sigma }}) \le \overline{\varepsilon },e_{CRE}(\overline{\psi };\tilde{\epsilon \hspace{-2.5pt}{\epsilon }},\sigma \hspace{-5.0pt}{\sigma }) \le \overline{\varepsilon }, (\tilde{\epsilon \hspace{-2.5pt}{\epsilon }},\tilde{\sigma \hspace{-5.0pt}{\sigma }}) \in \textbf{s}_{exp} \right\} } \end{aligned}$$

where the tolerance $\overline{\varepsilon }$ satisfies $\overline{\varepsilon } \ge \overline{E}_{CRE}(\overline{\psi })/|\Omega |$, and the local CRE is defined as:

28 $$\begin{aligned} { e^2_{CRE}(\overline{\psi };\epsilon \hspace{-2.5pt}{\epsilon },\sigma \hspace{-5.0pt}{\sigma }) = \overline{\psi }(\epsilon \hspace{-2.5pt}{\epsilon }) + \overline{\psi }^*(\sigma \hspace{-5.0pt}{\sigma }) - \text {Tr}[\sigma \hspace{-5.0pt}{\sigma }\epsilon \hspace{-2.5pt}{\epsilon }] } \end{aligned}$$

The experimental “points” should be sufficiently numerous and scattered so that the validation domain $\overline{\textbf{e}}$ contains a large ball of $\textbf{R}^6$ centered at 0.

#### Computation of the data-driven model

The problem to get $\overline{\psi } \in \uppsi$ being nonlinear, the computation method is iterative. At iteration $n+1$, there are two stages:

1. *K*-*S*-stage at iteration $n+1$ From the previous iterations, the following quantities have been computed: (i) $\psi _n \in \uppsi$; (ii) optimal fields $\left( \underline{U}_n^K(i),\sigma \hspace{-5.0pt}{\sigma }_n^S(i)\right) \in \varvec{\mathcal {U}}^K_{ad}(i) \times \varvec{\mathcal {S}}^S_{ad}(i)$, $i \in \textbf{N}_{test}$, computed from $\psi _{n-1}\in \uppsi$. The *K*-*S*-stage consists in the computation of the solution of each *i*-problem, $i \in N_{test}$, using the constitutive relation related to the convex function $\psi _n$. As we have seen in the previous section, this stage does not involve any serious difficulty for standard ill-posed computational problems; the classical finite element method can be used. In fact, we minimize the CRE for given $\psi _n$ to get optimal admissible fields:
  29 $$\begin{aligned} {\left( \underline{U}_{n+1}^K(i),\sigma \hspace{-5.0pt}{\sigma }_{n+1}^S(i)\right) } = \mathop {\mathrm {arg\,min}}\limits _{{\left( \tilde{\underline{U}}^K(i),\tilde{\sigma \hspace{-5.0pt}{\sigma }}^S(i)\right) } \in \varvec{\mathcal {U}}^K_{ad}(i) \times \varvec{\mathcal {S}}^S_{ad}(i)}E^2_{CRE}\left( \psi _n; {\tilde{\underline{U}}^K(i),\tilde{\sigma \hspace{-5.0pt}{\sigma }}^S(i)}\right) \end{aligned}$$
  and one has:
  30 $$\begin{aligned} \frac{1}{N_{test}}\sum _{i \in \textbf{N}_{test}} E^2_{CRE}\left( \psi _n; {\underline{U}_{n+1}^K(i),\sigma \hspace{-5.0pt}{\sigma }_{n+1}^S(i)}\right) \le \frac{1}{N_{test}}\sum _{i \in \textbf{N}_{test}} E^2_{CRE}\left( (\psi _n; {\underline{U}_n^K(i),\sigma \hspace{-5.0pt}{\sigma }_n^S(i)}\right) \end{aligned}$$
  The first term denoted $\overline{E}^2_{CRE}(\psi _n)$ can be interpreted as the CRE associated to the material model defined by $\psi _n \in \uppsi$. One has:
  31 $$\begin{aligned} \overline{E}^2_{CRE}(\psi _n) \le \overline{E}^2_{CRE}(\psi _{n-1}) \end{aligned}$$
  which indicates that the CRE decreases with the number of iterations. The iterative process is stopped when a stationary point is approximately reached. The final error value $\overline{E}_{CRE}(\overline{\psi })$ characterizes the quality of the computed data-driven material model.
2. $\psi$-stage at iteration $n+1$ From the previous stage, one can define:
  32 $$\begin{aligned} \psi \in \uppsi \longmapsto \overline{E}^2_{n+1}(\psi ) \equiv \frac{1}{N_{test}}\sum _{i \in \textbf{N}_{test}} E^2_{CRE}\left( (\psi ; {\underline{U}_{n+1}^K(i),\sigma \hspace{-5.0pt}{\sigma }_{n+1}^S(i)}\right) \end{aligned}$$
  and $\psi _{n+1} \in \uppsi$ is thus computed as:
  33 $$\begin{aligned} \psi _{n+1} = \mathop {\mathrm {arg\,min}}\limits _{\psi \in \uppsi } \overline{E}^2_{n+1}(\psi ) \end{aligned}$$
  This corresponds here to a convex regression problem that is to find a convex function belonging to $\uppsi$ which fits the best possible the data, question which is detailed in the next section.

#### Back to the $\psi$-stage at iteration $n+1$

Convex regression is an old question with still a lot of research works nowadays. A first set of works refers to pioneer papers [34, 69] where the convexity is obtained thanks to inequality constraints which are numerous and thus difficult to take into account. The detailed theory can be found in [51]. Among the numerous works in this direction, one can mention [4, 5, 65] and their references. Another set of works introduces the convex function being sought a priori, as a combination of elementary convex functions. Among these works, one can mention [30, 61, 74]. Input Convex Neural Networks (ICNN), introduced in [1] and widely used since then, follow the same representation of convex functions.

We propose here to follow [74] where the convex function being sought is described as the envelop of quasi-affine convex functions. Using the Voigt notation where strain and stress tensors ($\epsilon \hspace{-2.5pt}{\epsilon }$, $\sigma \hspace{-5.0pt}{\sigma }$) are represented by vectors ($\underline{\epsilon }$, $\underline{\sigma }$) of $\textbf{R}^6$, the sought potential $\psi$ is represented as:

34 $$\begin{aligned} \psi (\underline{\epsilon }) = \max _{j \in \textbf{J}}\psi _j(\underline{\epsilon }) ; \quad \psi _j(\underline{\epsilon }) = a_j + \underline{A}_j \cdot (\underline{\epsilon }- \underline{\epsilon }_j) + \frac{\tau }{2}(\underline{\epsilon }-\underline{\epsilon }_j)^T \mathbb {K}(\underline{\epsilon }-\underline{\epsilon }_j) \end{aligned}$$

The positive symmetric matrix $\mathbb {K}$ and the curvature $\tau$ are given. For $j \in \textbf{J}$, $(a_j,\underline{A}_j)$ and $\underline{\epsilon }_j$ are parameters. $\underline{\epsilon }_j$ are part of the discretization points of the strain space.

The advantage of the representation (34), apart from the fact that it can be visualized, is that its Legendre-Fenchel transform can be defined explicitly; one has:

35 $$\begin{aligned} \psi ^*(\underline{\sigma }) = \sup _{\underline{\epsilon }\in \textbf{R}^6}\left[ \underline{\sigma }^T\underline{\epsilon }- \psi (\underline{\epsilon })\right] = \min _{j \in \textbf{J}}\psi _j^*(\underline{\sigma }) \end{aligned}$$

with

36 $$\begin{aligned} \psi _j^*(\underline{\sigma }) = - a_j + \underline{\sigma }^T \underline{\epsilon }_j + \frac{1}{2\tau } (\underline{\sigma }-\underline{A}_j)^T \mathbb {K}^{-1}(\underline{\sigma }-\underline{A}_j) \end{aligned}$$

To belong to $\uppsi$, $\psi$ should in particular satisfy to:

37 $$\begin{aligned} \psi (\underline{0})=0 ; \quad {\frac{\partial \psi }{\partial \underline{\epsilon }}(\underline{0})} = \underline{0} \end{aligned}$$

The last relation being equivalent to $\psi (\underline{0})+\psi ^*(\underline{0})=0$, one has:

38 $$\begin{aligned} \begin{aligned}&{\max _{j \in \textbf{J}}}\left[ a_j - \underline{A}_j \cdot \underline{\epsilon }_j + \frac{\tau }{2}\underline{\epsilon }_j^T \mathbb {K}\underline{\epsilon }_j\right] = 0 \\&{\min _{j \in \textbf{J}}}\left[ -a_j + \frac{1}{2\tau }\underline{A}_j^T \mathbb {K}^{-1}\underline{A}_j\right] = 0 \end{aligned} \end{aligned}$$

One also remarks that under the constraints (37):

39 $$\begin{aligned} \forall \underline{\epsilon }\in \textbf{R}^6, \quad \psi (\underline{\epsilon }) + \psi ^*(\underline{0}) - 0 = \psi (\underline{\epsilon }) \ge 0 \end{aligned}$$

and consequently (38) are the only constraints defining $\psi$.

It follows that the function $\overline{E}_{n+1}(\psi )$ introduced in (32) is explicitly defined in terms of the parameters $(a_j,\underline{A}_j,\underline{\epsilon }_j)$ and consequently the problem to solve is a classical regression problem for which numerous numerical techniques are available. Of course, the optimal data-driven material model may be non-unique; consequently, a regularization (Lasso or Ridge) is welcome especially if the volume of data is relatively small.

### Particular case of linear materials

For linear elastic materials, it is remarkable that the minimization condition of the CRE functional can be written explicitly. Using the Voigt notation, the energy reads:

40 $$\begin{aligned} \psi (\epsilon \hspace{-2.5pt}{\epsilon }) = \frac{1}{2}\underline{\epsilon }^T \mathbb {K}\underline{\epsilon }\end{aligned}$$

where

41 $$\begin{aligned} \mathbb {K}\in \varvec{\mathcal {K}}\equiv \left\{ \mathbb {K}\; \text {matrix}\, (6,6) \;\text {s.t.}\; \mathbb {K}= \mathbb {K}^T, \mathbb {K}> 0 \right\} \end{aligned}$$

Here, the material model is thus defined by the Hooke tensor, the goal being to recover the optimal one.

The optimal $\mathbb {K}$ is the minimizer over $\varvec{\mathcal {K}}$ of the CRE for the family of tested structures; one has following (24) and (26):

42 $$\begin{aligned} \overline{E}^2_{CRE}(\mathbb {K}) = \frac{1}{N_{test}}\sum _{i \in \textbf{N}_{test}} \min _{\left( \tilde{\underline{U}}^K(i),\tilde{\sigma \hspace{-5.0pt}{\sigma }}^S(i)\right) \in \varvec{\mathcal {U}}^K_{ad}(i) \times \varvec{\mathcal {S}}^S_{ad}(i)} E^2_{CRE}\left( \mathbb {K}; \tilde{\underline{U}}^K(i),\tilde{\sigma \hspace{-5.0pt}{\sigma }}^S(i)\right) \end{aligned}$$

Denoting by $\underline{\epsilon }^K$ and $\underline{\sigma }^S$ the vectors corresponding respectively (in the Voigt notation) to optimal admissible fields $\epsilon \hspace{-2.5pt}{\epsilon }(\underline{U}^K)$ and $\sigma \hspace{-5.0pt}{\sigma }^S$, computed for given $\mathbb {K}$ and therefore implicitly depending on $\mathbb {K}$, one has:

43 $$\begin{aligned} \overline{E}^2_{CRE}(\mathbb {K}) = {\int _{\Omega }\left( \frac{1}{2}\text {Tr}[\mathbb {K}\mathbb {Q}^K] + \frac{1}{2}\text {Tr}[\mathbb {K}^{-1}\mathbb {Q}^S] - \frac{1}{N_{test}}\sum _{i \in \textbf{N}_{test}} \underline{\sigma }^S(i)^T\underline{\epsilon }^K(i)\right) \text {d}\Omega } \end{aligned}$$

with

44 $$\begin{aligned} \mathbb {Q}^K = \frac{1}{N_{test}}\sum _{i \in \textbf{N}_{test}} \underline{\epsilon }^K(i)\underline{\epsilon }^K(i)^T ; \quad \mathbb {Q}^S = \frac{1}{N_{test}}\sum _{i \in \textbf{N}_{test}} \underline{\sigma }^S(i) \underline{\sigma }^S(i)^T \end{aligned}$$

The differential of $\overline{E}_{CRE}$ should be equal to zero, which yields (using $\delta \mathbb {K}= -\mathbb {K}\delta \mathbb {K}^{-1}\mathbb {K}$):

45 $$\begin{aligned} 0= &   -\frac{1}{2}\text {Tr}[\delta \mathbb {K}^{-1}\mathbb {K}\mathbb {Q}^K\mathbb {K}] + \frac{1}{2}\text {Tr}[\delta \mathbb {K}^{-1}\mathbb {Q}^S]\nonumber \\  &   + \left[ \frac{1}{2}\text {Tr}[\mathbb {K}\delta \mathbb {Q}^K] + \frac{1}{2}\text {Tr}[\mathbb {K}^{-1}\delta \mathbb {Q}^S] - \delta \frac{1}{N_{test}}\sum _{i \in \textbf{N}_{test}}\underline{\sigma }^S(i)\underline{\epsilon }^K(i)^T\right] \end{aligned}$$

One can note that the last term between brackets is equal to zero because $(\underline{\epsilon }^K(i),\underline{\sigma }^S(i))$ is the minimizer of the CRE for the *i*-problem. Finally, one gets:

#### Property 3

For linear elastic materials, the minimization condition of the CRE is:

46 $$\begin{aligned} \mathbb {K}\mathbb {Q}^K \mathbb {K}= \mathbb {Q}^S ; \quad \mathbb {K}\in \varvec{\mathcal {K}}\end{aligned}$$

Let us come back to the $\psi$-stage at iteration $n+1$. One has:

47 $$\begin{aligned} \mathbb {K}\mathbb {Q}^K_{n+1}\mathbb {K}= \mathbb {Q}^S_{n+1} ; \quad \mathbb {K}\in \varvec{\mathcal {K}}\end{aligned}$$

so that

48 $$\begin{aligned} \left( \left[ \mathbb {Q}^K_{n+1}\right] ^{1/2} \mathbb {K}\left[ \mathbb {Q}^K_{n+1}\right] ^{1/2}\right) \left( \left[ \mathbb {Q}^K_{n+1}\right] ^{1/2} \mathbb {K}\left[ \mathbb {Q}^K_{n+1}\right] ^{1/2}\right) = \left[ \mathbb {Q}^K_{n+1}\right] ^{1/2} \mathbb {Q}_{n+1}^S \left[ \mathbb {Q}^K_{n+1}\right] ^{1/2}\nonumber \\ \end{aligned}$$

Consequently, if $\mathbb {Q}_{n+1}^K$ and $\mathbb {Q}_{n+1}^S$ are strictly positive:

49 $$\begin{aligned} \mathbb {K}^{n+1} = \left[ \mathbb {Q}^K_{n+1}\right] ^{-1/2} \left( \left[ \mathbb {Q}^K_{n+1}\right] ^{1/2} \mathbb {Q}_{n+1}^S \left[ \mathbb {Q}^K_{n+1}\right] ^{1/2}\right) ^{1/2}\left[ \mathbb {Q}^K_{n+1}\right] ^{-1/2} \end{aligned}$$

The case where matrices $\mathbb {Q}_{n+1}^K$ and $\mathbb {Q}_{n+1}^S$ are singular i.e., when the strain and stress spaces are not fully explored by the tests, is more complicated. We will omit the index $n+1$. Let $\mathbb {\pi }_K$ and $\mathbb {\pi }_S$ be the orthogonal projectors on the kernels of $\mathbb {Q}^K$ and $\mathbb {Q}^S$, respectively. To define the part of $\mathbb {K}$ which is not defined by the minimization condition, we use a regularization associated to the reference Hooke tensor $\mathbb {K}_0$ (strictly positive).

One remarks first from (48) that:

50 $$\begin{aligned} \Vert \mathbb {Q}^{K^{1/2}}\mathbb {K}\mathbb {Q}^{K^{1/2}}\underline{W}\Vert ^2 = \underline{W}^T \mathbb {Q}^{K^{1/2}} \mathbb {Q}^S \mathbb {Q}^{K^{1/2}} \underline{W}\end{aligned}$$

If $(\mathbb {I}-\mathbb {\pi }_K) \underline{W}\in Ker (\mathbb {Q}^S)$, the part $(\mathbb {I}-\mathbb {\pi }_K)\mathbb {K}(\mathbb {I}-\mathbb {\pi }_K)$ of the operator $\mathbb {K}$ is singular; one has:

51 $$\begin{aligned} { (\mathbb {I}-\mathbb {\pi }_K)\mathbb {K}(\mathbb {I}-\mathbb {\pi }_K) = \mathbb {Q}^{K^{-1/2}}\left[ \mathbb {Q}^{K^{1/2}} \mathbb {Q}^S \mathbb {Q}^{K^{1/2}}\right] ^{1/2}\mathbb {Q}^{K^{-1/2}} } \end{aligned}$$

To get a regular operator, let us consider the orthogonal projection $(\mathbb {I}-\overline{\mathbb {\pi }}_K)$ on the subspace $\text {Ker}^\perp (\mathbb {Q}^K) \cap \text {Ker}(\mathbb {Q}^S)$; one takes to get a non-singular $\mathbb {K}$:

52 $$\begin{aligned} { (\mathbb {I}-\mathbb {\pi }_K)\mathbb {K}(\mathbb {I}-\mathbb {\pi }_K) = \mathbb {Q}^{K^{-1/2}}\left[ \mathbb {Q}^{K^{1/2}} \mathbb {Q}^S \mathbb {Q}^{K^{1/2}}\right] ^{1/2}\mathbb {Q}^{K^{-1/2}} + (\mathbb {I}-\overline{\mathbb {\pi }}_K)\mathbb {K}_0(\mathbb {I}-\overline{\mathbb {\pi }}_K) } \end{aligned}$$

Moreover, one has:

53 $$\begin{aligned} { (\mathbb {I}-\mathbb {\pi }_K)\mathbb {K}\mathbb {\pi }_K = \mathbb {Q}^{K^{-1}}\left[ (\mathbb {I}-\mathbb {\pi }_K)\mathbb {K}(\mathbb {I}-\mathbb {\pi }_K)\right] ^{-1} \mathbb {Q}^S \mathbb {\pi }_K } \end{aligned}$$

To end, we take:

54 $$\begin{aligned} { \mathbb {\pi }_K \mathbb {K}\mathbb {\pi }_K = \mathbb {\pi }_K \mathbb {K}_0 \mathbb {\pi }_K } \end{aligned}$$

### Comparison with the pioneer data-driven approach

Let us consider the very particular situation where strain and stress are uniform for the family $\textbf{N}_{test}$ of tested structures. It follows that one can easily define the Experimental Constitutive Manifold $\varvec{\Gamma }_{exp}$:

55 $$\begin{aligned} { \varvec{\Gamma }_{exp} = \left\{ \left( \epsilon \hspace{-2.5pt}{\epsilon }^K(i),\sigma \hspace{-5.0pt}{\sigma }^S(i)\right) \in \textbf{R}^6 \times \textbf{R}^6, i \in \textbf{N}_{test}\right\} } \end{aligned}$$

The pioneer work [41] starts with the Experimental Constitutive Manifold which is in general not explicitly defined in terms of experimental data. To get the data-driven material model, an interpolation operator is added, for instance using a nearest neighbors (k-NN) algorithm.The present work is different in the sense that from materials science, the available mathematical shapes of the Experimental Constitutive Manifold are given.

For linear elastic materials, $\varvec{\Gamma }_{exp}$ should be close to a linear manifold that is computed thanks to the CRE. One gets for tests covering the strain and stress spaces:

56 $$\begin{aligned} { \mathbb {K}= [\mathbb {Q}^K]^{-1/2} \left( [\mathbb {Q}^K]^{1/2} \mathbb {Q}^S [\mathbb {Q}^K]^{1/2}\right) ^{1/2}[\mathbb {Q}^K]^{-1/2} } \end{aligned}$$

with

57 $$\begin{aligned} { \mathbb {Q}^K = \frac{1}{N_{test}}\sum _{i \in \textbf{N}_{test}} \underline{\epsilon }^K(i)\underline{\epsilon }^K(i)^T ; \quad \mathbb {Q}^S = \frac{1}{N_{test}}\sum _{i \in \textbf{N}_{test}} \underline{\sigma }^S(i) \underline{\sigma }^S(i)^T } \end{aligned}$$

In the case of nonlinear but stable elastic materials, $\varvec{\Gamma }_{exp}$ is nonlinear and should be close to a manifold defined by the convex potential $\psi \in \uppsi$. On has by minimizing the CRE:

58 $$\begin{aligned} { \overline{\psi } = \mathop {\mathrm {arg\,inf}}\limits _{\psi \in \uppsi } \frac{1}{N_{test}}\sum _{i \in \textbf{N}_{test}}\left[ \psi (\epsilon \hspace{-2.5pt}{\epsilon }^K(i)) + \psi ^*(\sigma \hspace{-5.0pt}{\sigma }^S(i)) - \text {Tr}[\sigma \hspace{-5.0pt}{\sigma }^S(i)\epsilon \hspace{-2.5pt}{\epsilon }^K(i)]\right] } \end{aligned}$$

## Data-driven material modeling—stable elasto-(visco-)plastic materials

The CRE-method is now set up for the data-driven modeling of stable elasto-(visco-)plastic materials. There are two key questions for which a solution is given:

- the mathematical shape of the most general data-driven model compatible with knowledge from physics and materials science;
- the computation of standard ill-posed problems for elasto-(visco-)plastic materials.

### Experimental data

We use similar notations as in the previous sections, but introducing now the time domain. We have a series of tested structures $i \in \textbf{N}_{test}$ over the time interval [0, *T*]. Data obtained from measurements is:

59 $$\begin{aligned} \underline{U}_d(i) \; \text {over}\, \partial _1 \Omega \times [0,T] ; \quad \underline{F}_d(i) \; \text {over}\, \partial _2 \Omega \times [0,T] \end{aligned}$$

The *i*-problems ($i \in \textbf{N}_{test}$) are standard ill-posed problems for which the admissible spaces for the displacement-stress pair $(\underline{U}^K,\sigma \hspace{-5.0pt}{\sigma }^S)$ are denoted $\varvec{\mathcal {U}}_{ad}^{K,[0,T]}$ and $\varvec{\mathcal {S}}_{ad}^{S,[0,T]}$.

### Mathematical shape of data-driven models

The goal in this section is to derive the mathematical shape of the most general available model, the elastic stiffness being known. We use here the internal variables framework which is very general and common in materials science; this is the basis of the material modeling developed here for stable elasto-(visco-)plastic materials.

#### The different internal variables

The state of the material at time *t* is defined by the value of the following set:

60 $$\begin{aligned} (\epsilon \hspace{-2.5pt}{\epsilon }_p,\textbf{X},\sigma \hspace{-5.0pt}{\sigma },\textbf{Y}) \end{aligned}$$

where $\epsilon \hspace{-2.5pt}{\epsilon }_p$ ($=\epsilon \hspace{-2.5pt}{\epsilon }-\textbf{K}^{-1}\sigma \hspace{-5.0pt}{\sigma }$) and $\sigma \hspace{-5.0pt}{\sigma }$ are observable variables, while the pair $(\textbf{X},\textbf{Y})$ gathers hidden internal variables. $\textbf{Y}$ is the force associated to $\textbf{X}$ and both belong to $\textbf{R}^q$. In a data-driven mathematical model, $\textbf{X}$ and $\textbf{Y}$ are a priori unknown as well as their dimension *q*.

#### The classical formulation

For the sake of simplicity, one assumes isothermal conditions. Following the first principle of thermodynamics of irreversible processes, we consider a Helmholtz free energy *e* which depends on the state of the material defined by the elastic strain $\epsilon \hspace{-2.5pt}{\epsilon }_e$, the hidden variable $\textbf{X}\in \textbf{R}^q$ and the cumulated inelastic strain *p*:

61 $$\begin{aligned} e(\epsilon \hspace{-2.5pt}{\epsilon }_e,\textbf{X},p) \end{aligned}$$

$\textbf{X}$ has often a precise interpretation; for example, in the case of kinematic hardening, $\textbf{X}$ is associated in the classical models to the center of the elasticity domain.

As classically done in materials science, one introduces the following decoupling hypothesis (H$_1$) used in this section:

62 $$\begin{aligned} e = \frac{1}{2}\text {Tr}[\textbf{K}\epsilon \hspace{-2.5pt}{\epsilon }_e \epsilon \hspace{-2.5pt}{\epsilon }_e] + \psi (\textbf{X},p) \end{aligned}$$

It follows the state equations defining the forces associated to $(\epsilon \hspace{-2.5pt}{\epsilon }_e,\textbf{X},p)$:

63 $$\begin{aligned} \sigma \hspace{-5.0pt}{\sigma }= \textbf{K}\epsilon \hspace{-2.5pt}{\epsilon }_e ; \quad \textbf{Y}= \frac{\partial \psi }{\partial \textbf{X}} ; \quad R = \frac{\partial \psi }{\partial p} \end{aligned}$$

The dissipation is equal to:

64 $$\begin{aligned} \begin{aligned} \omega&= \text {Tr}[\sigma \hspace{-5.0pt}{\sigma }\dot{\epsilon \hspace{-2.5pt}{\epsilon }}] - \dot{e} \\&= \text {Tr}[\sigma \hspace{-5.0pt}{\sigma }\dot{\epsilon \hspace{-2.5pt}{\epsilon }}_p] - \dot{\psi } \\&= \text {Tr}[\sigma \hspace{-5.0pt}{\sigma }\dot{\epsilon \hspace{-2.5pt}{\epsilon }}_p] - \textbf{Y}\cdot \dot{\textbf{X}} - R \dot{p} \end{aligned} \end{aligned}$$

The second principle of thermodynamics of irreversible processes indicates that the dissipation $\omega$ is positive or null for all admissible histories of the material. To satisfy this constraint, one adds the state evolution laws:

65 $$\begin{aligned} \left[ \begin{array}{c}\dot{\epsilon \hspace{-2.5pt}{\epsilon }}_p \\ -\dot{\textbf{X}} \\ -\dot{p} \end{array} \right] = \textbf{B}\left( \left[ \begin{array}{c}\sigma \hspace{-5.0pt}{\sigma }\\ \textbf{Y}\\ R \end{array} \right] \right) \end{aligned}$$

with $\epsilon \hspace{-2.5pt}{\epsilon }_p=0$, $\textbf{X}=0$ and $p=0$ at $t=0$, and $\textbf{B}$ is positive.

#### The normal formulation

The hidden internal variables $(\textbf{X},\textbf{Y}) \in \textbf{R}^q \times \textbf{R}^q$ are not intrinsic in the sense that another pair $(\textbf{X}',\textbf{Y}') \in \textbf{R}^q \times \textbf{R}^q$ can lead to the same observable quantities i.e. the same material model. This legitimates (as done in [47]) a choice more remarkable than the others, the so-called normal formulation. It results from a transformation of hidden variables, from $(\textbf{X},\textbf{Y})$ to $(\tilde{\textbf{X}},\tilde{\textbf{Y}})$, which leads to an equivalence between the kinematic and static variables, of the form:

66 $$\begin{aligned} \tilde{\textbf{X}} = \varvec{\Lambda }\tilde{\textbf{Y}} \end{aligned}$$

where $\varvec{\Lambda }$ is a positive symmetric constant linear operator. Here, we take $\varvec{\Lambda }= \textbf{I}$. This formulation is not unique, and the following property introduces a particular one where the only assumption is the decoupling hypothesis (H$_1$). Moreover, this normal formulation preserves the energy and the dissipation.

##### Property 4

Let the Helmholtz free energy be:

67 $$\begin{aligned} e(\epsilon \hspace{-2.5pt}{\epsilon }_e,\textbf{X},p) = \frac{1}{2}\text {Tr}[\textbf{K}\epsilon \hspace{-2.5pt}{\epsilon }_e \epsilon \hspace{-2.5pt}{\epsilon }_e] + \psi (\textbf{X},p) \end{aligned}$$

where $\psi$ is a convex positive function, twice differentiable such that:

68 $$\begin{aligned} \psi (\textbf{0},0)=0 ; \quad \frac{\partial \psi }{\partial \textbf{X}}(\textbf{0},0)=\textbf{0} ; \quad \frac{\partial \psi }{\partial p}(\textbf{0},0)=0 \end{aligned}$$

There is an internal variables transform:

69 $$\begin{aligned} \tilde{\textbf{X}}=\textbf{Q}_v(\textbf{X},p) ; \quad \tilde{p} = q_v(\textbf{X},p) \end{aligned}$$

such that the energy can be written with the new variables $(\tilde{\textbf{X}},\tilde{p})$:

70 $$\begin{aligned} e = \frac{1}{2}\text {Tr}[\textbf{K}\epsilon \hspace{-2.5pt}{\epsilon }_e \epsilon \hspace{-2.5pt}{\epsilon }_e] + \frac{1}{2}\tilde{\textbf{X}} \cdot \tilde{\textbf{X}} + \frac{1}{2}\tilde{p}^2 \end{aligned}$$

The dissipation is also preserved. The force $\tilde{\textbf{Y}}$ associated to $\tilde{\textbf{X}}$ is such that $\tilde{\textbf{Y}}=\tilde{\textbf{X}}$ and $\tilde{p}$ can be taken in $\textbf{R}^+$.

##### Proof

Let $$\textbf{Z}=\left[ \begin{array}{c}\textbf{X}\\ p\end{array}\right] \in \textbf{R}^{q+1}$$ and

71 $$\begin{aligned} \overline{\psi }(\textbf{Z}) = \textbf{Z}^T\left[ \int _0^1 \text {d}\mu \int _0^1 \lambda \text {d}\lambda \frac{\partial ^2 \psi }{\partial \textbf{Z}^2}(\lambda \mu \textbf{Z})\right] \textbf{Z}\end{aligned}$$

First, we prove that $\overline{\psi }(\textbf{Z})=\psi (\textbf{Z})$. One has:

72 $$\begin{aligned} \overline{\psi }(\textbf{Z}) = \int _0^1 \text {d}\mu \frac{\text {d}}{\text {d}\mu } \Gamma (\mu ) \end{aligned}$$

with $\Gamma (\mu ) = \int _0^1 \text {d}\lambda \frac{\partial \psi }{\partial \textbf{Z}}(\mu \lambda \textbf{Z}) \textbf{Z}$. It follows:

73 $$\begin{aligned} \overline{\psi }(\textbf{Z}) = \Gamma (1) - \Gamma (0) = \int _0^1 \text {d}\lambda \frac{\partial \psi }{\partial \textbf{Z}}(\lambda \textbf{Z}) \textbf{Z}\end{aligned}$$

Finally, one has:

74 $$\begin{aligned} \overline{\psi }(\textbf{Z}) = \psi (\textbf{Z}) - \psi (\textbf{0}) = \psi (\textbf{Z}) \end{aligned}$$

Let us introduce the operator

75 $$\begin{aligned} \textbf{A}= 2 \int _0^1\int _0^1 \lambda \text {d}\lambda \text {d}\mu \frac{\partial ^2\psi }{\partial \textbf{Z}^2}(\lambda \mu \textbf{Z}) \end{aligned}$$

It is symmetric and also positive, $\psi$ being a convex function. Consequently, one can define the operator $\textbf{A}^{1/2}$ and the new state variables are:

76 $$\begin{aligned} \left[ \begin{array}{c}\tilde{\textbf{X}}\\ \tilde{p}\end{array}\right] = \textbf{A}^{1/2} \left[ \begin{array}{c}\textbf{X}\\ p\end{array}\right] \end{aligned}$$

with $$\psi (\tilde{\textbf{X}},\tilde{p}) = \frac{1}{2}\tilde{\textbf{X}}\cdot \tilde{\textbf{X}} + \frac{1}{2}\tilde{p}^2$$.

The energy being preserved, it is also true for the dissipation. The operator $\textbf{B}$ which defines the state evolution laws is of course modified, the relation between the observable variables being preserved. $\square$

The normal formulation has serious advantages. For stable materials with a convex energy, it allows to divide by two the number of hidden internal variables and to get an energy parametrized by the Hooke tensor $\textbf{K}$ alone.

Elasto-viscoplasticity is characterized by the fact that the components of the generalized strain rate, i.e. $\dot{\epsilon \hspace{-2.5pt}{\epsilon }}_p$, $\dot{\tilde{\textbf{X}}}$, $\dot{\tilde{p}}$, are null at the same time. Consequently, the most general mathematical model can be written:

77 $$\begin{aligned} \begin{aligned} \dot{\epsilon \hspace{-2.5pt}{\epsilon }}_p&= \textbf{g}(\sigma \hspace{-5.0pt}{\sigma },\tilde{\textbf{X}},\tilde{p})\dot{\tilde{p}} \\ \dot{\tilde{\textbf{X}}}&= \textbf{h}(\sigma \hspace{-5.0pt}{\sigma },\tilde{\textbf{X}},\tilde{p})\dot{\tilde{p}} \\ \dot{\tilde{p}}&= f(\sigma \hspace{-5.0pt}{\sigma },\tilde{\textbf{X}},\tilde{p}) \ge 0 \\ \epsilon \hspace{-2.5pt}{\epsilon }_p&= 0, \tilde{\textbf{X}} = \textbf{0}, \tilde{p} = 0 \; \text {at} \, t=0 \end{aligned} \end{aligned}$$

This model is defined by three functions and the number *q* of hidden variables. To fulfill the second principle of thermodynamics of irreversible processes, one should satisfy:

78 $$\begin{aligned} \text {Tr}[\sigma \hspace{-5.0pt}{\sigma }\dot{\epsilon \hspace{-2.5pt}{\epsilon }}_p] - \tilde{\textbf{X}} \cdot \dot{\tilde{\textbf{X}}} - \tilde{p}.\dot{\tilde{p}} \ge 0 \end{aligned}$$

For elastoplastic materials for which the behavior is independent of the loading rate, the third equation of (77) is replaced by:

79 $$\begin{aligned} \dot{\tilde{p}} \overline{f}(\sigma \hspace{-5.0pt}{\sigma },\tilde{\textbf{X}} \tilde{p}) = 0 ; \quad \dot{\tilde{p}}\ge 0 ; \quad \overline{f} \le 0 \end{aligned}$$

##### Remark 2

We have shown in [50] that the functions $\textbf{g}$, $\textbf{h}$, *f* should be single-valued to get a consistent material model with (77) and (78). This univocity property is the basis of the construction method of the hidden internal state variables from data introduced in [24, 50]. Its interest is its generality. However, we will see that the CRE enables to solve the same problem with a more robust approach but less general in the sense that a CRE must be defined beforehand, constraint that we are considering today as relatively weak.

#### The subfamily of standard materials

The decoupling hypothesis (H$_1$) and the convexity property of the energy *e* belong to commonly accepted assumptions in materials science as they have been validated by experiments for stable materials. Consequently, they will be systematically taken into account a priori in the following. Here, we go one step further considering stable materials which, for most of them, can be described by a standard material model. Such a family, very common in materials science, has been validated for numerous materials. It has been introduced by B. Halphen and Q.S. Nguyen [29].

Precisely, a standard material model is defined by two potentials $\phi ^*(\sigma \hspace{-5.0pt}{\sigma },\tilde{\textbf{X}},\tilde{p})$ and $\phi (\dot{\epsilon \hspace{-2.5pt}{\epsilon }}_p,-\dot{\tilde{\textbf{X}}},-\dot{\tilde{p}})$ which are two dual convex functions related to the dissipation bilinear form:

80 $$\begin{aligned} \left( (\dot{\epsilon \hspace{-2.5pt}{\epsilon }}_p,-\dot{\tilde{\textbf{X}}},-\dot{\tilde{p}}), (\sigma \hspace{-5.0pt}{\sigma },\tilde{\textbf{X}},\tilde{p})\right) \in \textbf{R}^{6+q+1} \times \textbf{R}^{6+q+1} \longmapsto \text {Tr}[\sigma \hspace{-5.0pt}{\sigma }\dot{\epsilon \hspace{-2.5pt}{\epsilon }}_p]- \tilde{\textbf{X}}\cdot \dot{\tilde{\textbf{X}}} - \tilde{p}.\dot{\tilde{p}} \end{aligned}$$

One should also satisfy the second principle of thermodynamics of irreversible processes, that corresponds to:

81 $$\begin{aligned} {\phi ^*}(0,\textbf{0},0) = 0 ; \quad \phi ^{*} \ge 0 \end{aligned}$$

Such a material model depends only on one convex function and it is compatible with the two principles of thermodynamics. It also depends on the dimension *q* of the vector of hidden internal variables.

The local CRE then reads:

82 $$\begin{aligned} e^2_{CRE} = \phi (\dot{\epsilon \hspace{-2.5pt}{\epsilon }}_p,-\dot{\tilde{\textbf{X}}},-\dot{\tilde{p}}) + \phi ^*(\sigma \hspace{-5.0pt}{\sigma },\tilde{\textbf{X}},\tilde{p}) - \text {Tr}[\sigma \hspace{-5.0pt}{\sigma }\dot{\epsilon \hspace{-2.5pt}{\epsilon }}_p]+ \tilde{\textbf{X}}\cdot \dot{\tilde{\textbf{X}}} + \tilde{p}.\dot{\tilde{p}} \end{aligned}$$

and the global CRE over the time-space domain $[0,T] \times \Omega$ is equal to:

83 $$\begin{aligned} E^2_{CRE} = \int _0^T \text {d}t\int _{\Omega }\text {d}\Omega \eta (t) e^2_{CRE} \end{aligned}$$

where the weight $\eta (t)$ may be chosen as 1 or $(1-t/T)$.

Let us return to the normal formulation and the following question: is there a normal formulation for a standard initial model that retains the property of being standard? The answer has been given in [47]; one has:

##### Property 5

A standard material model defined by the convex potential ${\phi ^*}(\sigma \hspace{-5.0pt}{\sigma },\textbf{Z})$ and the inelastic part of the energy $\psi (\textbf{Z})$ admits a normal standard formulation is there exists an operator $\textbf{R}$ (defining a new set of hidden internal variables) such that:

84 $$\begin{aligned} \begin{aligned}&\tilde{\textbf{Z}} \equiv \left[ \begin{array}{c}\tilde{\textbf{X}}\\ \tilde{p}\end{array}\right] = \textbf{R}(\textbf{Z}) \\&\left( \frac{\partial \tilde{\textbf{Z}}}{\partial \textbf{Z}}\right) ^T\left( \frac{\partial \tilde{\textbf{Z}}}{\partial \textbf{Z}}\right) = \frac{\partial }{\partial \textbf{Z}}\left( \frac{\partial \psi }{\partial \textbf{Z}}\right) ^T \\&{\phi ^*}\left( \sigma \hspace{-5.0pt}{\sigma },\left( \frac{\partial \psi }{\partial \textbf{Z}}\right) ^T(\textbf{R}^{-1}(\tilde{\textbf{Z}}))\right) \; \text {is a convex function} \end{aligned} \end{aligned}$$

This normal formulation is different from the one introduced in “The normal formulation” section. The conditions of Property 5 are much more common than they appear at first. They are satisfied by classical parametrized models of materials science [47]; an illustration is given a little further on.

One can remark that the main condition:

85 $$\begin{aligned} \left( \frac{\partial \tilde{\textbf{Z}}}{\partial \textbf{Z}}\right) ^T\left( \frac{\partial \tilde{\textbf{Z}}}{\partial \textbf{Z}}\right) = \frac{\partial }{\partial \textbf{Z}}\left( \frac{\partial \psi }{\partial \textbf{Z}}\right) ^T \end{aligned}$$

involves a deformation. The right-hand side should be positive which implies the convexity of the energy. An approximation can be given if we neglect the square of the nonlinear part of $\textbf{R}(\textbf{Z})$ denoted $\textbf{R}^*(\textbf{Z})$; one has:

86 $$\begin{aligned} \tilde{\textbf{Z}} = \textbf{R}(\textbf{Z}) = \textbf{A}_0 \textbf{Z}+ \textbf{R}^*(\textbf{Z}) \end{aligned}$$

It follows with $\varvec{\Delta }= \frac{\partial }{\partial \textbf{Z}}\textbf{R}^*(\textbf{Z})$:

87 $$\begin{aligned} { \textbf{A}^T_0 \varvec{\Delta }+ \varvec{\Delta }^T \textbf{A}_0 + \textbf{A}_0^T \textbf{A}_0 = \frac{\partial }{\partial \textbf{Z}}\left( \frac{\partial \psi }{\partial \textbf{Z}}\right) ^T} \end{aligned}$$

One gets the operator $\textbf{R}$ as:

88 $$\begin{aligned} { \tilde{\textbf{Z}} = \frac{1}{2}\left[ \left( \textbf{A}_0^{-1}\right) ^T \left( \frac{\partial \psi }{\partial \textbf{Z}}\right) ^T + \textbf{A}_0 \textbf{Z}\right] } \end{aligned}$$

which shows that if we neglect the square of the nonlinear part of $\frac{\partial \psi }{\partial \textbf{Z}})^T$, the material model is both normal and standard.

Finally, as a conclusion of the previous developments, we consider that the family of normal and standard models provides a reasonable framework for the data-driven modeling of stable elasto-(visco-)plastic materials.

***A classical model in materials science***

Let us consider first the standard version of the Chaboche-Marquis elasto-(visco-)plastic model (see [47, 52]). We will see that this model admits a version which is both standard and normal.

The material is supposed to be isotropic; the internal hidden variable is a second order tensor $\mathbb {X}$ with $\text {Tr}[\mathbb {X}]=0$, its associated force being $\mathbb {Y}$ with $\text {Tr}[\mathbb {Y}]=0$. The dissipation potential in viscoplasticity is with classical notations:

89 $$\begin{aligned} {\phi ^*}(\sigma \hspace{-5.0pt}{\sigma }_D,\mathbb {Y},R) = \frac{k}{n+1}\langle z \rangle _+^{n+1} \quad n >0 \end{aligned}$$

with

90 $$\begin{aligned} \begin{aligned} z&= |\sigma \hspace{-5.0pt}{\sigma }_D-\mathbb {Y}| + \frac{1}{2}a|\mathbb {Y}|^2 - R \\ \mathbb {Y}&= c \mathbb {X}\\ R&= \gamma (p) \\ p&= \int _0^T |\dot{\epsilon \hspace{-2.5pt}{\epsilon }}_p| \text {d}t\end{aligned} \end{aligned}$$

*k*, *n*, *a*, *c* are positive material parameters. The threshold function:

91 $$\begin{aligned} p \in \textbf{R}^+ \longmapsto \gamma (p) \in \textbf{R}^+ \end{aligned}$$

is also a material function which is supposed to be concave and increasing.

To get a normal standard formulation, let us introduce:

92 $$\begin{aligned} \begin{aligned} \tilde{\mathbb {X}}&= c^{1/2} \mathbb {X}\\ \tilde{p}&= \int _0^p \text {d}p [\frac{\partial \gamma }{\partial p}]^{1/2} \\ R&= \ell (\tilde{p}) \end{aligned} \end{aligned}$$

One has ${\tilde{\phi }^*}(\sigma \hspace{-5.0pt}{\sigma }_D,\tilde{\mathbb {X}},\tilde{p})={\phi ^*}(\sigma \hspace{-5.0pt}{\sigma }_D,c^{1/2} \mathbb {X},\ell (\tilde{p}))$ and one can check that ${\tilde{\phi }^*}$ is convex.

***Standard material model for elastoplastic materials***

Let us consider the case of elastoplastic materials. The potential $\phi ^*$ is the indicator function of the elastic domain which is convex; one has:

93 $$\begin{aligned} \phi ^*(\sigma \hspace{-5.0pt}{\sigma },\tilde{\textbf{X}},\tilde{p})= {\left\{ \begin{array}{ll} 0 \qquad \text {if}\; \overline{f}(\sigma \hspace{-5.0pt}{\sigma },\tilde{\textbf{X}},\tilde{p}) \le 0 \\ + \infty \quad \text {otherwise} \end{array}\right. } \end{aligned}$$

with $$\overline{f}(0,0,0) \le 0$$.

This mathematical model depends also on one convex function $\overline{f}$. This function can be simplified because we know from materials science that the elasticity domain grows with $\tilde{p}$ which can be interpreted as a material time, i.e.:

94 $$\begin{aligned} \overline{f}_{,\tilde{p}} < 0 \quad (\text {H}_2) \end{aligned}$$

It follows the property:

##### Property 6

Under the (H$_2$) hypothesis and if $\overline{f}$ is convex and piecewise regular, $\overline{f}$ can be replaced by:

95 $$\begin{aligned} a(\sigma \hspace{-5.0pt}{\sigma },\tilde{\textbf{X}}) - \tilde{p} \end{aligned}$$

where *a* is a convex function such that $a(0,\textbf{0})\le 0$.

##### Proof

From $\overline{f}(\sigma \hspace{-5.0pt}{\sigma },\tilde{\textbf{X}},\tilde{p})=0$, one gets

96 $$\begin{aligned} \tilde{p} = a(\sigma \hspace{-5.0pt}{\sigma },\tilde{\textbf{X}}) \end{aligned}$$

and we have:

97 $$\begin{aligned} \overline{f}(\sigma \hspace{-5.0pt}{\sigma },\tilde{\textbf{X}},a(\sigma \hspace{-5.0pt}{\sigma },\tilde{\textbf{X}}))=0 \end{aligned}$$

The second differential of $\overline{f}(\sigma \hspace{-5.0pt}{\sigma },\tilde{\textbf{X}},a(\sigma \hspace{-5.0pt}{\sigma },\tilde{\textbf{X}}))$ is:

98 $$\begin{aligned} 0 = [\delta ^2 \overline{f}](\sigma \hspace{-5.0pt}{\sigma },\tilde{\textbf{X}},\tilde{p})_{\tilde{p}=a(\sigma \hspace{-5.0pt}{\sigma },\tilde{\textbf{X}})} + \frac{\partial f}{\partial p}[\delta ^2 a](\sigma \hspace{-5.0pt}{\sigma },\tilde{\textbf{X}}) \end{aligned}$$

It follows $\delta ^2 a \ge 0$ and consequently the convexity of *a*. Moreover, from $\overline{f}(0,\textbf{0},0)\le 0$, $p \ge 0$, one gets:

99 $$\begin{aligned} a(0,\textbf{0}) \le 0 \end{aligned}$$

Finally, the data-driven mathematical model compatible with the knowledge from physics and materials science is defined by the convex function *a* alone, such that $a(0,\textbf{0}) \le 0$. $\square$

##### Remark 3

Some more assumptions can be introduced for particular materials. For example, for metallic materials, the plastic incompressibility is commonly used. Isotropy or orthotropy properties can also be added and allow important complexity reduction.

It follows that the dual potential $\phi$ is:

100 $$\begin{aligned} \begin{aligned} \phi (\dot{\epsilon \hspace{-2.5pt}{\epsilon }}_p,-\dot{\tilde{\textbf{X}}},-\dot{\tilde{p}})&= \sup _{\sigma \hspace{-5.0pt}{\sigma },\tilde{\textbf{X}},\tilde{p},a(\sigma \hspace{-5.0pt}{\sigma },\tilde{\textbf{X}}) \le \tilde{p}}\left[ \text {Tr}[\sigma \hspace{-5.0pt}{\sigma }\dot{\epsilon \hspace{-2.5pt}{\epsilon }}_p] - \tilde{\textbf{X}}\cdot \dot{\tilde{\textbf{X}}} - \tilde{p}.\dot{\tilde{p}} \right] \\&= \sup _{\sigma \hspace{-5.0pt}{\sigma },\tilde{\textbf{X}},\tilde{p},a(\sigma \hspace{-5.0pt}{\sigma },\tilde{\textbf{X}}) \le \tilde{p}}\left[ \dot{\tilde{p}}(\text {Tr}[\sigma \hspace{-5.0pt}{\sigma }\dot{\epsilon \hspace{-2.5pt}{\epsilon }}_p/\dot{\tilde{p}}] - \tilde{\textbf{X}}\cdot \dot{\tilde{\textbf{X}}}/\dot{\tilde{p}}-a(\sigma \hspace{-5.0pt}{\sigma },\tilde{\textbf{X}})+\dot{\tilde{p}}(a-\tilde{p}) \right] \qquad \\&= \dot{\tilde{p}} a^*(\dot{\epsilon \hspace{-2.5pt}{\epsilon }}_p/\dot{\tilde{p}},-\dot{\tilde{\textbf{X}}}/\dot{\tilde{p}}) + 0 \end{aligned} \end{aligned}$$

The local CRE can be written:

101 $$\begin{aligned} e^2_{CRE} = \dot{\tilde{p}} \left[ a^*(\dot{\epsilon \hspace{-2.5pt}{\epsilon }}_p/\dot{\tilde{p}},-\dot{\tilde{\textbf{X}}}/\dot{\tilde{p}}) + a(\sigma \hspace{-5.0pt}{\sigma },\tilde{\textbf{X}}) - \text {Tr}[\sigma \hspace{-5.0pt}{\sigma }\dot{\epsilon \hspace{-2.5pt}{\epsilon }}_p/\dot{\tilde{p}}] + \tilde{\textbf{X}} \cdot \dot{\tilde{\textbf{X}}}/\dot{\tilde{p}}\right] + \dot{\tilde{p}}\left[ \tilde{p} - a(\sigma \hspace{-5.0pt}{\sigma },\tilde{\textbf{X}})\right] \nonumber \\ \end{aligned}$$

where the quantities between brackets are positive or null.

***Standard material model for elasto-viscoplastic materials***

A common assumption in materials science is to consider viscoplasticity as an extension or regularization of plasticity. The elastic domain being described by the convex function *a*, the potential is defined as:

102 $$\begin{aligned} {\phi ^*}(\sigma \hspace{-5.0pt}{\sigma },\tilde{\textbf{X}},\tilde{p}) = \gamma (\langle a(\sigma \hspace{-5.0pt}{\sigma },\tilde{\textbf{X}})-\tilde{p}\rangle _+) \end{aligned}$$

where $\gamma$ is an increasing convex function defined over $\textbf{R}^+$ such that $\gamma (0)=0$. The potential ${\phi ^*}$ is thus convex, positive and satisfies:

103 $$\begin{aligned} {\phi ^*}(0,\textbf{0},0) = 0 \end{aligned}$$

To get the dual potential ${\phi }$, one has to take the Legendre-Fenchel transform of a combination of convex functions named composite convex functions for which results are given in [36]. Here, one has:

104 $$\begin{aligned} \begin{aligned} {\phi }(\dot{\epsilon \hspace{-2.5pt}{\epsilon }}_p,-\dot{\tilde{\textbf{X}}},-\dot{\tilde{p}})&= \sup _{\sigma \hspace{-5.0pt}{\sigma },\tilde{\textbf{X}},\tilde{p} \in \textbf{R}^{6+q} \times \textbf{R}^+} \left[ \text {Tr}[\sigma \hspace{-5.0pt}{\sigma }\dot{\epsilon \hspace{-2.5pt}{\epsilon }}_p] - \tilde{\textbf{X}}\cdot \dot{\tilde{\textbf{X}}} - \tilde{p}.\dot{\tilde{p}} - \gamma (\langle a(\sigma \hspace{-5.0pt}{\sigma },\tilde{\textbf{X}})-\tilde{p}\rangle _+)\right] \\&= \sup _{\sigma \hspace{-5.0pt}{\sigma },\tilde{\textbf{X}},\tilde{p} \in \textbf{R}^{6+q} \times \textbf{R}^+} \left[ (\dot{\tilde{p}}(a(\sigma \hspace{-5.0pt}{\sigma },\tilde{\textbf{X}})-\tilde{p}) - \gamma (\langle a(\sigma \hspace{-5.0pt}{\sigma },\tilde{\textbf{X}})-\tilde{p}\rangle _+)) \right. \\&\quad \left. + \dot{\tilde{p}}(a^*(\dot{\epsilon \hspace{-2.5pt}{\epsilon }}_p/\dot{\tilde{p}},-\dot{\tilde{\textbf{X}}}/\dot{\tilde{p}}) + a(\sigma \hspace{-5.0pt}{\sigma },\tilde{\textbf{X}}) - \text {Tr}[\sigma \hspace{-5.0pt}{\sigma }\dot{\epsilon \hspace{-2.5pt}{\epsilon }}_p/\dot{\tilde{p}}] + \tilde{\textbf{X}}\cdot \dot{\tilde{\textbf{X}}}/\dot{\tilde{p}})\right] \\&= \gamma ^*(\dot{\tilde{p}}) + \dot{\tilde{p}}a^*(\dot{\epsilon \hspace{-2.5pt}{\epsilon }}_p/\dot{\tilde{p}},-\dot{\tilde{\textbf{X}}}/\dot{\tilde{p}}) \end{aligned} \end{aligned}$$

It follows that the local CRE is equal to:

105 $$\begin{aligned} \begin{aligned} e^2_{CRE} =\,&\dot{\tilde{p}}\left[ a(\sigma \hspace{-5.0pt}{\sigma },\tilde{\textbf{X}}) + a^*(\dot{\epsilon \hspace{-2.5pt}{\epsilon }}_p/\dot{\tilde{p}},-\dot{\tilde{\textbf{X}}}/\dot{\tilde{p}}) - \text {Tr}[\sigma \hspace{-5.0pt}{\sigma }\dot{\epsilon \hspace{-2.5pt}{\epsilon }}_p/\dot{\tilde{p}}] + \tilde{\textbf{X}}\cdot \dot{\tilde{\textbf{X}}}/\dot{\tilde{p}} \right] \\&+ \left[ \gamma ^*(\dot{\tilde{p}}) + \gamma (\langle a(\sigma \hspace{-5.0pt}{\sigma },\tilde{\textbf{X}}) - \tilde{p}\rangle _+) - \dot{\tilde{p}} \langle a(\sigma \hspace{-5.0pt}{\sigma },\tilde{\textbf{X}})-\tilde{p}\rangle _+ \right] \\&+ \left[ \dot{\tilde{p}} \langle a(\sigma \hspace{-5.0pt}{\sigma },\tilde{\textbf{X}})-\tilde{p}\rangle _- \right] \end{aligned} \end{aligned}$$

Let us note that the three quantities between brackets are positive or null.

***Illustration: back to the normal/standard model of Chaboche-Marquis***

We have seen that the potential ${\phi ^*}(\sigma \hspace{-5.0pt}{\sigma }_D,\tilde{\mathbb {X}},\tilde{p})$ is equal to:

106 $$\begin{aligned} {\phi ^*}(\sigma \hspace{-5.0pt}{\sigma }_D,\tilde{\mathbb {X}},\tilde{p}) = \frac{k}{n+1}\langle z \rangle _+^{n+1} \end{aligned}$$

with

107 $$\begin{aligned} z = |\sigma \hspace{-5.0pt}{\sigma }_D - c^{1/2}\tilde{\mathbb {X}}| + \frac{1}{2}ac|\tilde{\mathbb {X}}|^2 - \ell (\tilde{p}) \end{aligned}$$

It follows:

108 $$\begin{aligned} a(\sigma \hspace{-5.0pt}{\sigma }_D,\tilde{\mathbb {X}}) = \ell ^{-1}(|\sigma \hspace{-5.0pt}{\sigma }_D - c^{1/2}\tilde{\mathbb {X}}| + \frac{1}{2}ac|\tilde{\mathbb {X}}|^2) \end{aligned}$$

which is convex because $\ell$ is an increasing concave function. One also has:

109 $$\begin{aligned} {\phi ^*}(\sigma \hspace{-5.0pt}{\sigma }_D,\tilde{\mathbb {X}},\tilde{p}) = \gamma (\langle a(\sigma \hspace{-5.0pt}{\sigma }_D,\tilde{\mathbb {X}})-\tilde{p}\rangle _+) \end{aligned}$$

with

110 $$\begin{aligned} \gamma (r) = \frac{k}{n+1}[\ell (r)]^{n+1} \quad \forall r \in \textbf{R}^+ \end{aligned}$$

### Optimal solution of standard ill-posed problems

Let us consider that the behavior of the material is known and defined by the potential ${\phi ^*}(\sigma \hspace{-5.0pt}{\sigma },\tilde{\mathbb {X}},\tilde{p})$ which is a convex function such that:

111 $$\begin{aligned} {\phi ^*} \ge 0 ; \quad {\phi ^*}(0,\textbf{0},0) = 0 \end{aligned}$$

The displacement $\underline{U}^K$ and the stress $\sigma \hspace{-5.0pt}{\sigma }^S$ defined over $[0,T] \times \Omega$ should be admissible, i.e.:

112 $$\begin{aligned} \underline{U}^K \in \varvec{\mathcal {U}}^{K,[0,T]}_{ad} ; \quad \sigma \hspace{-5.0pt}{\sigma }^S \in \varvec{\mathcal {S}}^{S,[0,T]}_{ad} \end{aligned}$$

and the problem to solve is a standard ill-posed problem. A solution could be given by the pair $(\underline{U}^K,\sigma \hspace{-5.0pt}{\sigma }^S)$ which minimizes the global CRE functional $E_{CRE}$ over the space $\varvec{\mathcal {U}}^{K,[0,T]}_{ad} \times \varvec{\mathcal {S}}^{S,[0,T]}_{ad}$. $E_{CRE}$ being a strongly convex function for standard material models, the solution is unique. Here, the optimal solution is computed.

First we construct a solution defined by a modified potential ${\phi ^*_M}$ and in a second time, we prove that it is optimal. For elasto-(visco-)plastic materials, the modified constitutive relation is also elasto-(visco-)plastic but nonlocal; however, the optimal solution can be computed without difficulty thanks to an incremental numerical technique.

#### A remarkable constraint

We will see that for standard ill-posed problems, $\dot{\epsilon \hspace{-2.5pt}{\epsilon }}_p$ is not a priori arbitrary. One has:

113 $$\begin{aligned} \dot{\epsilon \hspace{-2.5pt}{\epsilon }}_p = \dot{\epsilon \hspace{-2.5pt}{\epsilon }}^K - \textbf{K}^{-1}\dot{\sigma \hspace{-5.0pt}{\sigma }}^S \end{aligned}$$

and the question is to compute $\epsilon \hspace{-2.5pt}{\epsilon }^K$ and $\sigma \hspace{-5.0pt}{\sigma }^S$ from $\dot{\epsilon \hspace{-2.5pt}{\epsilon }}_p$. First, let us compute $\sigma \hspace{-5.0pt}{\sigma }^S$; it is defined for $t \in ]0,T[$ by:

114 $$\begin{aligned} \sigma \hspace{-5.0pt}{\sigma }^S \in \varvec{\mathcal {S}}_{ad}^S \;; \; \int _{\Omega }\text {Tr}[\sigma \hspace{-5.0pt}{\sigma }^S \textbf{K}^{-1}\sigma \hspace{-5.0pt}{\sigma }^*] \text {d}\Omega = - \int _{\Omega }\text {Tr}[(\epsilon \hspace{-2.5pt}{\epsilon }_p-\epsilon \hspace{-2.5pt}{\epsilon }_e^K)\sigma \hspace{-5.0pt}{\sigma }^*]\text {d}\Omega \quad \forall \sigma \hspace{-5.0pt}{\sigma }^*\in \varvec{\mathcal {S}}^S_{ad,0} \end{aligned}$$

where $\epsilon \hspace{-2.5pt}{\epsilon }_e^K$ is the elastic solution for the *K*-data:

115 $$\begin{aligned} \underline{U}_d \; \text {over}\, \partial _1 \Omega \times ]0,T[ ; \quad \underline{F}_d \; \text {over}\, (\partial _2 \Omega - \partial _{12}\Omega ) \times ]0,T[ ; \quad \underline{f}_d = 0 \end{aligned}$$

It follows:

116 $$\begin{aligned} \epsilon \hspace{-2.5pt}{\epsilon }_p + \textbf{K}^{-1}\sigma \hspace{-5.0pt}{\sigma }^S = \epsilon \hspace{-2.5pt}{\epsilon }(\underline{U}) \end{aligned}$$

with $\underline{U}\in [\textbf{H}^1(\Omega )]^3$ and $\underline{U}=\underline{U}_d$ over $(\partial _1 \Omega - \partial _{12}\Omega )$.

To get $\underline{U}^K = \underline{U}$, $\underline{U}$ should also satisfy:

117 $$\begin{aligned} \underline{U}= \underline{U}_d \; \text {over}\, \partial _{12}\Omega \end{aligned}$$

which is equivalent to:

118 $$\begin{aligned} \int _{\Omega }\text {Tr}[\textbf{K}\epsilon \hspace{-2.5pt}{\epsilon }(\underline{U})\epsilon \hspace{-2.5pt}{\epsilon }(\underline{W}^*)]\text {d}\Omega = \int _{\partial _{12}\Omega } \textbf{K}\epsilon \hspace{-2.5pt}{\epsilon }(\underline{W}^*)\underline{n}\cdot \underline{U}_d \text {d}S\quad \forall \underline{W}^*\in \varvec{\mathcal {W}}\end{aligned}$$

with

119 $$\begin{aligned}  &   \varvec{\mathcal {W}}\equiv \left\{ \underline{U}\in [\textbf{H}^1(\Omega )]^3 \; \text {s.t.} \; \underline{\text {div\,}}(\textbf{K}\epsilon \hspace{-2.5pt}{\epsilon }(\underline{U}))=0 \; \text {over}\, \Omega , \underline{U}_{|\partial _1 \Omega - \partial _{12}\Omega } = 0,\right. \nonumber \\  &   \qquad \qquad \left. \textbf{K}\epsilon \hspace{-2.5pt}{\epsilon }(\underline{U})\underline{n}_{|\partial _2 \Omega - \partial _{12}\Omega } = 0 \right\} \end{aligned}$$

Using (116), we get from (118) the following property:

##### Property 7

For standard ill-posed problems, the inelastic strain satisfies the constraint:

120 $$\begin{aligned} \int _{\Omega }\text {Tr}[\textbf{K}\epsilon \hspace{-2.5pt}{\epsilon }_p \epsilon \hspace{-2.5pt}{\epsilon }(\underline{W}^*)]\text {d}\Omega = \int _{\Omega }\text {Tr}[\textbf{K}\epsilon \hspace{-2.5pt}{\epsilon }_e^{KS}\epsilon \hspace{-2.5pt}{\epsilon }(\underline{W}^*)]\text {d}\Omega \quad \forall \underline{W}^*\in \varvec{\mathcal {W}}\end{aligned}$$

where $\epsilon \hspace{-2.5pt}{\epsilon }_e^{KS} \equiv \epsilon \hspace{-2.5pt}{\epsilon }_e^K - \epsilon \hspace{-2.5pt}{\epsilon }_e^S$, $\epsilon \hspace{-2.5pt}{\epsilon }_e^K$ and $\epsilon \hspace{-2.5pt}{\epsilon }_e^S$ being the elastic solutions for the *K*-data and the *S*-data, respectively.

##### Remark 4

For a well-posed computational problem, the constraint (120) disappears.

#### Modified potential ${\Phi ^*_M}$

Let us introduce the global potentials:

121 $$\begin{aligned} {\Phi ^*}(\sigma \hspace{-5.0pt}{\sigma },\tilde{\textbf{X}},\tilde{p}) = \int _{\Omega }{\phi ^*}(\sigma \hspace{-5.0pt}{\sigma },\tilde{\textbf{X}},\tilde{p})\text {d}\Omega ; \quad {\Phi }(\dot{\epsilon \hspace{-2.5pt}{\epsilon }}_p,-\dot{\tilde{\textbf{X}}},-\dot{\tilde{p}}) = \int _{\Omega }{\phi }(\dot{\epsilon \hspace{-2.5pt}{\epsilon }}_p,-\dot{\tilde{\textbf{X}}},-\dot{\tilde{p}}) \text {d}\Omega \end{aligned}$$

One considers first the case where the reference potential ${\Phi ^*}$ is regular. The $E_{CRE}$-minimization leads for fixed $(\sigma \hspace{-5.0pt}{\sigma },\tilde{\textbf{X}},\tilde{p})$ to the minimization of:

122 $$\begin{aligned} {\Phi }(\dot{\epsilon \hspace{-2.5pt}{\epsilon }}_p,-\dot{\tilde{\textbf{X}}},-\dot{\tilde{p}}) - \int _{\Omega }\left[ \text {Tr}[\sigma \hspace{-5.0pt}{\sigma }\dot{\epsilon \hspace{-2.5pt}{\epsilon }}_p] - \tilde{\textbf{X}}\cdot \dot{\tilde{\textbf{X}}} - \tilde{p}.\dot{\tilde{p}}\right] \text {d}\Omega \end{aligned}$$

with the constraint:

123 $$\begin{aligned} \int _{\Omega }\text {Tr}\left[ \textbf{K}(\dot{\epsilon \hspace{-2.5pt}{\epsilon }}_p-\dot{\epsilon \hspace{-2.5pt}{\epsilon }}_e^{KS})\epsilon \hspace{-2.5pt}{\epsilon }(\underline{W}^*)\right] \text {d}\Omega = 0 \quad \forall \underline{W}^*\in \varvec{\mathcal {W}}\end{aligned}$$

This constraint being equivalent to $\dot{\epsilon \hspace{-2.5pt}{\epsilon }}_p \in \textbf{H}_p$, the previous minimization problem can be rewritten as the minimization of:

124 $$\begin{aligned} {\Phi }(\dot{\epsilon \hspace{-2.5pt}{\epsilon }}_p,-\dot{\tilde{\textbf{X}}},-\dot{\tilde{p}}) + \upchi _{\textbf{H}_p}(\dot{\epsilon \hspace{-2.5pt}{\epsilon }}_p,-\dot{\tilde{\textbf{X}}},-\dot{\tilde{p}}) - \int _{\Omega }\left[ \text {Tr}[\sigma \hspace{-5.0pt}{\sigma }\dot{\epsilon \hspace{-2.5pt}{\epsilon }}_p] - \tilde{\textbf{X}}\cdot \dot{\tilde{\textbf{X}}} - \tilde{p}.\dot{\tilde{p}}\right] \text {d}\Omega \end{aligned}$$

where $\upchi _{\textbf{H}_p}$ is the characteristic function of the subspace $\textbf{H}_p$. It follows that $(\dot{\epsilon \hspace{-2.5pt}{\epsilon }}_p,-\dot{\tilde{\textbf{X}}},-\dot{\tilde{p}})$ is the subdifferential of the Legendre-Fenchel transform of ${\Phi } + \upchi _{\textbf{H}_p}$, for which we know ${\Phi ^*}$ and $\upchi ^*_{\textbf{H}_p}(\sigma \hspace{-5.0pt}{\sigma },\tilde{\textbf{X}},\tilde{p})$ equal to:

125 $$\begin{aligned} {\left\{ \begin{array}{ll} \text {Tr}[\sigma \hspace{-5.0pt}{\sigma }\dot{\epsilon \hspace{-2.5pt}{\epsilon }}_e^{KS}] \quad \text {if}\, \sigma \hspace{-5.0pt}{\sigma }= -\textbf{K}\epsilon \hspace{-2.5pt}{\epsilon }(\underline{W}) \; \text {with}\, \underline{W}\in \varvec{\mathcal {W}}\\ + \infty \quad \quad \qquad \text {otherwise} \end{array}\right. } \end{aligned}$$

This is a composite convex function; from [35], we get that the Legendre-Fenchel transform of ${\Phi } + \upchi _{\textbf{H}_p}$ is:

126 $$\begin{aligned} \inf _{\sigma \hspace{-5.0pt}{\sigma }=\sigma \hspace{-5.0pt}{\sigma }'-\textbf{K}\epsilon \hspace{-2.5pt}{\epsilon }(\underline{W}), \underline{W}\in \varvec{\mathcal {W}}}\left[ {\Phi ^*}(\sigma \hspace{-5.0pt}{\sigma }',\tilde{\textbf{X}},\tilde{p}) + \upchi ^*_{\textbf{H}_p}(-\textbf{K}\epsilon \hspace{-2.5pt}{\epsilon }(\underline{W}),\tilde{\textbf{X}},\tilde{p})\right] \end{aligned}$$

Finally, we have:

127 $$\begin{aligned} \inf _{\underline{W}\in \varvec{\mathcal {W}}} \left[ {\Phi ^*}(\sigma \hspace{-5.0pt}{\sigma }+ \textbf{K}\epsilon \hspace{-2.5pt}{\epsilon }(\underline{W}),\tilde{\textbf{X}},\tilde{p}) - \int _{\Omega }\text {Tr}[\textbf{K}\dot{\epsilon \hspace{-2.5pt}{\epsilon }}_e^{KS}\epsilon \hspace{-2.5pt}{\epsilon }(\underline{W})]\text {d}\Omega \right] \end{aligned}$$

which defines the modified potential ${\Phi ^*_M}$. In the case of elastoplastic materials, one has a slight modification:

128 $$\begin{aligned} {\Phi ^*_M}(\sigma \hspace{-5.0pt}{\sigma },\tilde{\textbf{X}},\tilde{p})= &   {\Phi ^*}(\sigma \hspace{-5.0pt}{\sigma },\tilde{\textbf{X}},\tilde{p})\nonumber \\  &   + \inf _{\underline{W}\in \varvec{\mathcal {W}}} \left[ {\Phi ^*}(\sigma \hspace{-5.0pt}{\sigma }+ \textbf{K}\epsilon \hspace{-2.5pt}{\epsilon }(\underline{W}),\tilde{\textbf{X}},\tilde{p}) - \int _{\Omega }\text {Tr}[\textbf{K}\dot{\epsilon \hspace{-2.5pt}{\epsilon }}_e^{KS}\epsilon \hspace{-2.5pt}{\epsilon }(\underline{W})]\text {d}\Omega \right] \end{aligned}$$

#### Properties of the solution

The considered solution is characterized by the two dual convex potentials ${\phi ^*_M}$ and ${\phi _M}$, which defines a nonlocal in space constitutive relation. However, this property does not involve any serious difficulty for the computation. The classical incremental method can be used, the only new point being the calculation at each time step of the $\underline{W}$-problem for which a reduction method is recommended.

To prove the uniqueness of the solution, let us consider two distinct solutions $(\sigma \hspace{-5.0pt}{\sigma },\tilde{\textbf{X}},\tilde{p})$ and $(\sigma \hspace{-5.0pt}{\sigma }',\tilde{\textbf{X}}',\tilde{p}')$. One has:

129 $$\begin{aligned} \begin{aligned} \dot{A}&\equiv \int _{\Omega }\left[ \text {Tr}[(\sigma \hspace{-5.0pt}{\sigma }-\sigma \hspace{-5.0pt}{\sigma }')(\dot{\epsilon \hspace{-2.5pt}{\epsilon }}_p-\dot{\epsilon \hspace{-2.5pt}{\epsilon }}'_p] - (\tilde{\textbf{X}}-\tilde{\textbf{X}}')\cdot (\dot{\tilde{\textbf{X}}}-\dot{\tilde{\textbf{X}}}') - (\tilde{p}-\tilde{p}').(\dot{\tilde{p}}-\dot{\tilde{p}}')\right] \text {d}\Omega \\&= {\phi '_M} + {\phi ^*_M} - \int _{\Omega }\left[ \text {Tr}[\sigma \hspace{-5.0pt}{\sigma }\dot{\epsilon \hspace{-2.5pt}{\epsilon }}'_p] - \tilde{\textbf{X}}\cdot \dot{\tilde{\textbf{X}}}' - \tilde{p}.\dot{\tilde{p}}' \right] \text {d}\Omega \\&\quad + {\phi _M} + {\phi ^{*'}_M} - \int _{\Omega }\left[ \text {Tr}[\sigma \hspace{-5.0pt}{\sigma }' \dot{\epsilon \hspace{-2.5pt}{\epsilon }}_p] - \tilde{\textbf{X}}'\cdot \dot{\tilde{\textbf{X}}} - \tilde{p}'.\dot{\tilde{p}} \right] \text {d}\Omega \ge 0 \end{aligned} \end{aligned}$$

Moreover, we have for standard ill-posed problems:

130 $$\begin{aligned} \int _{\Omega }\text {Tr}[(\sigma \hspace{-5.0pt}{\sigma }^S-\sigma \hspace{-5.0pt}{\sigma }^{S'})(\dot{\epsilon \hspace{-2.5pt}{\epsilon }}^K-\dot{\epsilon \hspace{-2.5pt}{\epsilon }}^{K'})]\text {d}\Omega = 0 \end{aligned}$$

and thus:

131 $$\begin{aligned} \int _{\Omega }\text {Tr}[(\sigma \hspace{-5.0pt}{\sigma }^S-\sigma \hspace{-5.0pt}{\sigma }^{S'})(\dot{\epsilon \hspace{-2.5pt}{\epsilon }}_p-\dot{\epsilon \hspace{-2.5pt}{\epsilon }}'_p)]\text {d}\Omega = - \int _{\Omega }\text {Tr}[(\sigma \hspace{-5.0pt}{\sigma }^S-\sigma \hspace{-5.0pt}{\sigma }^{S'})\textbf{K}^{-1}(\dot{\sigma \hspace{-5.0pt}{\sigma }}^S-\dot{\sigma \hspace{-5.0pt}{\sigma }}^{S'})]\text {d}\Omega \end{aligned}$$

It follows from (129), (131) and the initial conditions at $t=0$:

132 $$\begin{aligned} A_{|t}= &   -\frac{1}{2}\int _{\Omega }\left[ \text {Tr}[(\sigma \hspace{-5.0pt}{\sigma }^S-\sigma \hspace{-5.0pt}{\sigma }^{S'})\textbf{K}^{-1}(\sigma \hspace{-5.0pt}{\sigma }^S-\sigma \hspace{-5.0pt}{\sigma }^{S'})] \right. \nonumber \\  &   \left. + (\tilde{\textbf{X}}-\tilde{\textbf{X}}')\cdot (\tilde{\textbf{X}}-\tilde{\textbf{X}}') + (\tilde{p}-\tilde{p}')^2 \right] \text {d}\Omega \ge 0 \end{aligned}$$

Consequently, the two solutions can not be distinct which proves the uniqueness.

Moreover, we have the following main property:

##### Property 8

The solution associated to the modified potentials ${\Phi _M}$ and ${\Phi ^*_M}$ is optimal i.e. it minimizes the CRE.

##### Proof

Let $(\underline{U}^{K'},\sigma \hspace{-5.0pt}{\sigma }^{S'})$ be the optimal solution i.e., the minimizer of the global CRE over the admissible space $\varvec{\mathcal {U}}_{ad}^{K,[0,T]} \times \varvec{\mathcal {S}}_{ad}^{S,[0,T]}$ taking into account the constraint (120). One has the following result, where $\Phi$ can be replaced by $\Phi _M$:

133 $$\begin{aligned} a\equiv &   \int _0^T \eta (t) \text {d}t\left[ {\Phi _M}(\dot{\epsilon \hspace{-2.5pt}{\epsilon }}_p',-\dot{\tilde{\textbf{X}}}',-\dot{\tilde{p}}') + {\Phi ^*}(\sigma \hspace{-5.0pt}{\sigma }',\tilde{\textbf{X}}',\tilde{p}') \right. \nonumber \\  &   \left. - \int _{\Omega }\left[ \text {Tr}[\sigma \hspace{-5.0pt}{\sigma }'\dot{\epsilon \hspace{-2.5pt}{\epsilon }}_p'] -\tilde{\textbf{X}}'\cdot \dot{\tilde{\textbf{X}}}' - \tilde{p}'\dot{\tilde{p}}'\right] \text {d}\Omega \right] \end{aligned}$$

The solution $(\underline{U}^K,\sigma \hspace{-5.0pt}{\sigma }^S)$ associated to the modified constitutive relation satisfies:

134 $$\begin{aligned} 0= &   \int _0^T \eta (t) \text {d}t\left[ {\Phi _M}(\dot{\epsilon \hspace{-2.5pt}{\epsilon }}_p,-\dot{\tilde{\textbf{X}}},-\dot{\tilde{p}}) + {\Phi _M^*}(\sigma \hspace{-5.0pt}{\sigma },\tilde{\textbf{X}},\tilde{p}) \right. \nonumber \\  &   \left. - \int _{\Omega }\left[ \text {Tr}[\sigma \hspace{-5.0pt}{\sigma }\dot{\epsilon \hspace{-2.5pt}{\epsilon }}_p]-\tilde{\textbf{X}}\cdot \dot{\tilde{\textbf{X}}} - \tilde{p}\dot{\tilde{p}}\right] \text {d}\Omega \right] \end{aligned}$$

Let us compute:

135 $$\begin{aligned} \overline{A}= &   \int _0^T \eta (t) \text {d}t\int _{\Omega }\left[ \text {Tr}[(\sigma \hspace{-5.0pt}{\sigma }-\sigma \hspace{-5.0pt}{\sigma }')(\dot{\epsilon \hspace{-2.5pt}{\epsilon }}_p-\dot{\epsilon \hspace{-2.5pt}{\epsilon }}'_p)]\right. \nonumber \\  &   \left. -(\tilde{\textbf{X}}-\tilde{\textbf{X}}')\cdot (\dot{\tilde{\textbf{X}}}-\dot{\tilde{\textbf{X}}}') - (\tilde{p}-\tilde{p}').(\dot{\tilde{p}}-\dot{\tilde{p}}')\right] \text {d}\Omega \end{aligned}$$

One has from (133) and (134):

136 $$\begin{aligned} \begin{aligned} \overline{A} =&\int _0^T \eta (t) \text {d}t\left[ {\Phi _M}((\dot{\epsilon \hspace{-2.5pt}{\epsilon }}_p',-\dot{\tilde{\textbf{X}}}',-\dot{\tilde{p}}') + {\Phi _M^*}(\sigma \hspace{-5.0pt}{\sigma },\tilde{\textbf{X}},\tilde{p}) \right. \\&\left. - \int _{\Omega }\left[ \text {Tr}[\sigma \hspace{-5.0pt}{\sigma }\dot{\epsilon \hspace{-2.5pt}{\epsilon }}_p'] -\tilde{\textbf{X}}\cdot \dot{\tilde{\textbf{X}}}' - \tilde{p}\dot{\tilde{p}}'\right] \text {d}\Omega \right] \\&+ \int _0^T \eta (t) \text {d}t\left[ {\Phi _M}((\dot{\epsilon \hspace{-2.5pt}{\epsilon }}_p,-\dot{\tilde{\textbf{X}}},-\dot{\tilde{p}}) + {\Phi ^*}(\sigma \hspace{-5.0pt}{\sigma }',\tilde{\textbf{X}}',\tilde{p}') \right. \\&\left. - \int _{\Omega }\left[ \text {Tr}[\sigma \hspace{-5.0pt}{\sigma }'\dot{\epsilon \hspace{-2.5pt}{\epsilon }}_p] -\tilde{\textbf{X}}'\cdot \dot{\tilde{\textbf{X}}} - \tilde{p}'\dot{\tilde{p}}\right] \text {d}\Omega - a \right] \end{aligned} \end{aligned}$$

and consequently $\overline{A} \ge 0$. Moreover, we get:

137 $$\begin{aligned} \int _{\Omega }\text {Tr}[(\sigma \hspace{-5.0pt}{\sigma }-\sigma \hspace{-5.0pt}{\sigma }')(\dot{\epsilon \hspace{-2.5pt}{\epsilon }}-\dot{\epsilon \hspace{-2.5pt}{\epsilon }}')]\text {d}\Omega = 0 \end{aligned}$$

and thus:

138 $$\begin{aligned} \int _{\Omega }\text {Tr}[(\sigma \hspace{-5.0pt}{\sigma }-\sigma \hspace{-5.0pt}{\sigma }')(\dot{\epsilon \hspace{-2.5pt}{\epsilon }}_p-\dot{\epsilon \hspace{-2.5pt}{\epsilon }}_p')]\text {d}\Omega = -\int _{\Omega }\text {Tr}[(\sigma \hspace{-5.0pt}{\sigma }-\sigma \hspace{-5.0pt}{\sigma }')\textbf{K}^{-1}(\dot{\sigma \hspace{-5.0pt}{\sigma }}-\dot{\sigma \hspace{-5.0pt}{\sigma }}')]\text {d}\Omega \end{aligned}$$

It follows with $\eta (t) = 1-t/T$:

139 $$\begin{aligned} \overline{A}= &   -\frac{1}{2T} \int _0^T\text {d}t\int _{\Omega }\left[ \text {Tr}[(\sigma \hspace{-5.0pt}{\sigma }-\sigma \hspace{-5.0pt}{\sigma }')\textbf{K}^{-1}(\dot{\sigma \hspace{-5.0pt}{\sigma }}-\dot{\sigma \hspace{-5.0pt}{\sigma }}')] \right. \nonumber \\  &   \left. + (\tilde{\textbf{X}}-\tilde{\textbf{X}}')\cdot (\tilde{\textbf{X}}-\tilde{\textbf{X}}') + (\tilde{p}-\tilde{p}')^2 \right] \text {d}\Omega \end{aligned}$$

and thus $\overline{A} = 0$. One concludes that:

140 $$\begin{aligned} (\sigma \hspace{-5.0pt}{\sigma },\tilde{\textbf{X}},\tilde{p}) = (\sigma \hspace{-5.0pt}{\sigma }',\tilde{\textbf{X}}',\tilde{p}') \; \text {over}\, [0,T] \times \Omega \end{aligned}$$

Let us remind that $(\dot{\epsilon \hspace{-2.5pt}{\epsilon }}_p,-\dot{\tilde{\textbf{X}}},-\dot{\tilde{p}})$ is the minimizer of the CRE for fixed $(\sigma \hspace{-5.0pt}{\sigma },\tilde{\textbf{X}},\tilde{p})$. It follows:

141 $$\begin{aligned} (\dot{\epsilon \hspace{-2.5pt}{\epsilon }}_p,-\dot{\tilde{\textbf{X}}},-\dot{\tilde{p}}) = (\dot{\epsilon \hspace{-2.5pt}{\epsilon }}'_p,-\dot{\tilde{\textbf{X}}}',-\dot{\tilde{p}}') = \partial {\Phi ^*_M}(\sigma \hspace{-5.0pt}{\sigma },\tilde{\textbf{X}},\tilde{p}) \end{aligned}$$

$$\square $$

### Optimal data-driven material model for elastoplastic materials

We only consider the case of elastoplastic materials as elasto-viscoplastic materials can be treated in the same way. The only difference is the identification of one more function, the function $\gamma$.

#### Experimental data

Experimental data has been introduced in “Experimental data” section. It is, for $i \in \textbf{N}_{test}$:

142 $$\begin{aligned} \underline{U}_d(i) \; \; \text {over}\, \partial _1 \Omega \times [0,T] ; \quad \textbf{F}_d(i) \; \; \text {over}\, \partial _2 \Omega \times [0,T] \end{aligned}$$

The Hooke tensor has been identified beforehand.

We also have seen previously that for stable elastoplastic materials, the mathematical shape of the most general data-driven model compatible with knowledge from physics and materials science is defined by a unique convex function *a* belonging to $\textbf{A}$:

143 $$\begin{aligned} a \in \textbf{A}\equiv \left\{ a \; \text {s.t.} \; \text {{ a} is a convex function over}\, \textbf{R}^{6+q}, a(0,\textbf{0}) \le 0\right\} \end{aligned}$$

#### Computation of the optimal data-driven model

The optimal data-driven material model is the minimizer of the global CRE for the family of tested structures. One first has, for $i \in \textbf{N}_{test}$:

144 $$\begin{aligned} { \left( \underline{U}^K(i),\sigma \hspace{-5.0pt}{\sigma }^S(i)\right) = \mathop {\mathrm {arg\,min}}\limits _{\left( \tilde{\underline{U}}^K(i),\tilde{\sigma \hspace{-5.0pt}{\sigma }}^S(i)\right) \in \varvec{\mathcal {U}}^K_{ad}(i) \times \varvec{\mathcal {S}}^S_{ad}(i)}E^2_{CRE}\left( q,a;\tilde{\underline{U}}^K(i),\tilde{\sigma \hspace{-5.0pt}{\sigma }}^S(i)\right) } \end{aligned}$$

Then, after defining:

145 $$\begin{aligned} { \overline{E}^2_{CRE}(q,a) = \frac{1}{N_{test}}\sum _{i \in \textbf{N}_{test}}E^2_{CRE}\left( q,a;\underline{U}^K(i),\sigma \hspace{-5.0pt}{\sigma }^S(i)\right) } \end{aligned}$$

the optimal data-driven material model is recovered as:

146 $$\begin{aligned} { \boxed { (\overline{q},\overline{a}) = \mathop {\mathrm {arg\,inf}}\limits _{a \in \textbf{A}, q \in \textbf{N}^+}\overline{E}^2_{CRE}(q,a) }} \end{aligned}$$

The method to solve this nonlinear problem is similar to the one presented for solving nonlinear elastic material models; the only difference is related to the dimension *q* of the vector $\tilde{\textbf{X}}$ of hidden internal variables, which belongs to the unknowns of the optimization problem. Consequently, the optimization problem is first solved for a fixed value of *q*. If the final value of the error is too large, it may mean that the value of the dimension *q* needs to be increased. The optimal data-driven model should thus be recalculated, leading to a reduction of the error. If this reduction is sufficient, one stops the calculation.

##### Remark 5

Finding a convex function *a* defined over $\textbf{R}^{6+q}$ is a high-dimensional convex regression problem for which additional assumptions on the function *a* are more than welcome; they are mandatory to reduce the complexity of the regression problem. Isotropy or orthotropy properties can also be added, but probably the best and most common way is to use micro/meso information through the FE$^2$ method. Of course, the CRE as presented here can also be used to identify parametrized models from materials science. Nevertheless, to be considered data-driven, data should carry significant information in these approaches.

### Data-driven modeling of stable elasto-(visco-)plastic materials taking measurement noise into account

One considers stable materials described by standard models in the situation where the measurement noise should be taken into account. Following [71], the main source of noise comes from full-field displacement measurements. It is noteworthy that in the CRE, this noise impacts only the elastic strain rate $\dot{\epsilon \hspace{-2.5pt}{\epsilon }}_e^{KS}$ from the constraint:

147 $$\begin{aligned} \int _{\Omega }\text {Tr}[(\dot{\epsilon \hspace{-2.5pt}{\epsilon }}_p-\dot{\epsilon \hspace{-2.5pt}{\epsilon }}_e^{KS})\textbf{K}\epsilon \hspace{-2.5pt}{\epsilon }(\underline{W}^*)]\text {d}\Omega = 0 \quad \forall \underline{W}^*\in \varvec{\mathcal {W}}\end{aligned}$$

where $\dot{\epsilon \hspace{-2.5pt}{\epsilon }}_e^{KS}$ depends on the prescribed displacement $\underline{U}_d$. To take the measurement noise into account, we propose to satisfy this constraint approximately through a modified Constitutive Relation Error:

148 $$\begin{aligned} \begin{aligned} mE^2_{CRE} =\,&\int _0^T \eta (t)\left[ {\Phi }(\dot{\epsilon \hspace{-2.5pt}{\epsilon }}_p,-\dot{\tilde{\textbf{X}}},-\dot{\tilde{p}}) + {\Phi ^*}(\sigma \hspace{-5.0pt}{\sigma },\tilde{\textbf{X}},\tilde{p}) \right. \\&\left. - \int _{\Omega }\left[ \text {Tr}[\sigma \hspace{-5.0pt}{\sigma }\dot{\epsilon \hspace{-2.5pt}{\epsilon }}_p] - \tilde{\textbf{X}}\cdot \dot{\tilde{\textbf{X}}} - \tilde{p}.\dot{\tilde{p}}\right] \text {d}\Omega \right] \text {d}t\\&+ \int _0^T \frac{\mu }{2}|||\dot{\epsilon \hspace{-2.5pt}{\epsilon }}_p-\dot{\epsilon \hspace{-2.5pt}{\epsilon }}_e^{KS}|||^2 \text {d}t\end{aligned} \end{aligned}$$

where the weight $\mu$ is chosen such that $|||\dot{\epsilon \hspace{-2.5pt}{\epsilon }}_p-\dot{\epsilon \hspace{-2.5pt}{\epsilon }}_e^{KS}|||$ is of the order of magnitude of the measurement noise. Moreover, one has:

149 $$\begin{aligned} \int _{\Omega }\text {Tr}[\textbf{K}\epsilon \hspace{-2.5pt}{\epsilon }(\underline{W})\epsilon \hspace{-2.5pt}{\epsilon }(\underline{W})]\text {d}\Omega = |||\dot{\epsilon \hspace{-2.5pt}{\epsilon }}_p-\dot{\epsilon \hspace{-2.5pt}{\epsilon }}_e^{KS}|||^2 \end{aligned}$$

with $$\underline{W}\in \varvec{\mathcal {W}}$$ and

150 $$\begin{aligned} \int _{\Omega }\text {Tr}[\textbf{K}\epsilon \hspace{-2.5pt}{\epsilon }(\underline{W})\epsilon \hspace{-2.5pt}{\epsilon }(\underline{W}^*)]\text {d}\Omega = \int _{\Omega }\text {Tr}[(\dot{\epsilon \hspace{-2.5pt}{\epsilon }}_p-\dot{\epsilon \hspace{-2.5pt}{\epsilon }}_e^{KS})\textbf{K}\epsilon \hspace{-2.5pt}{\epsilon }(\underline{W}^*)]\text {d}\Omega \quad \forall \underline{W}^*\in \varvec{\mathcal {W}}\end{aligned}$$

Here, the modified CRE (mCRE) replaces the classical CRE developed in the previous sections.

#### The hybrid optimal solution

Following the “Modified potential ${\Phi ^*_M}$” section, one computes the dual function of ${\Phi _M}$ defined by:

151 $$\begin{aligned} {\Phi _M}(\dot{\epsilon \hspace{-2.5pt}{\epsilon }}_p,-\dot{\tilde{\textbf{X}}},-\dot{\tilde{p}}) = {\Phi }(\dot{\epsilon \hspace{-2.5pt}{\epsilon }}_p,-\dot{\tilde{\textbf{X}}},-\dot{\tilde{p}}) + \frac{\mu }{2}|||\dot{\epsilon \hspace{-2.5pt}{\epsilon }}_p-\dot{\epsilon \hspace{-2.5pt}{\epsilon }}_e^{KS}|||^2 \end{aligned}$$

First, let us determine the dual function of $\frac{\mu }{2}|||\dot{\epsilon \hspace{-2.5pt}{\epsilon }}_p-\dot{\epsilon \hspace{-2.5pt}{\epsilon }}_e^{KS}|||^2$; one has:

- if $$\sigma \hspace{-5.0pt}{\sigma }= -\textbf{K}\epsilon \hspace{-2.5pt}{\epsilon }(\underline{W})$$ with $$\underline{W}\in \varvec{\mathcal {W}}$$:
  152 $$\begin{aligned} g^*(\sigma \hspace{-5.0pt}{\sigma },\tilde{\textbf{X}},\tilde{p}) = \int _{\Omega }\left[ \frac{\mu }{2}\text {Tr}[\sigma \hspace{-5.0pt}{\sigma }\textbf{K}^{-1}\sigma \hspace{-5.0pt}{\sigma }] + \text {Tr}[\sigma \hspace{-5.0pt}{\sigma }\dot{\epsilon \hspace{-2.5pt}{\epsilon }}_e^{KS}]\right] \text {d}\Omega \end{aligned}$$
- otherwise:
  153 $$\begin{aligned} g^*(\sigma \hspace{-5.0pt}{\sigma },\tilde{\textbf{X}},\tilde{p}) = + \infty \end{aligned}$$

From [35], we get that the dual function ${\Phi ^*_M}$ is:

154 $$\begin{aligned} \inf _{\sigma \hspace{-5.0pt}{\sigma }=\sigma \hspace{-5.0pt}{\sigma }'-\textbf{K}\epsilon \hspace{-2.5pt}{\epsilon }(\underline{W})}\left[ {\Phi ^*}(\sigma \hspace{-5.0pt}{\sigma }',\tilde{\textbf{X}},\tilde{p}) + \int _{\Omega }\left[ \frac{1}{2\mu }\text {Tr}[\textbf{K}\epsilon \hspace{-2.5pt}{\epsilon }(\underline{W})\epsilon \hspace{-2.5pt}{\epsilon }(\underline{W})] - \text {Tr}[\textbf{K}\epsilon \hspace{-2.5pt}{\epsilon }(\underline{W})\dot{\epsilon \hspace{-2.5pt}{\epsilon }}_e^{KS}] \right] \text {d}\Omega \right] \end{aligned}$$

One can note that if $\mu = + \infty$, we get the potential associated to the constraint (147). Following the proof of Property 8, we can also prove that the solution which satisfies the modified constitutive relation (potentials ${\Phi _M}$ and ${\Phi _M^*}$) is optimal i.e. it is the minimizer of $mE_{CRE}$ over the admissible space $\varvec{\mathcal {U}}_{ad}^{K,[0,T]} \times \varvec{\mathcal {S}}_{ad}^{S,[0,T]}$.

##### Remark 6

The hybrid optimal solution can be useful in nonlinear computational mechanics to compute hybrid numerical twins.

#### The optimal data-driven model

All which is described in the “Optimal data-driven material model for elastoplastic materials” section is still valid.

## Conclusion

The CRE has been developed as a general tool to compute data-driven models and also classical parametrized models of materials science seen as minimizers of the global CRE. For stable materials described by a standard model, the normal formulation with only one convex function to identify was put forward. For a given reference constitutive relation, an optimal solution i.e. the admissible displacement-stress pair which minimizes the global CRE has been characterized by its modified constitutive relation, nonlocal in the space variable but easy to compute. Such a solution extended to noisy data leads to the easy computation of a hybrid numerical twin involving elasto-(visco-)plastic materials.

Let us note that materials defined by their microstructure for which a data-driven macromodel should be built is a potential range of application; a great advantage is the large volume of available data that facilitates the convex regression. Based on the homogenized theory of periodic media introduced in [73], this question has already been the subject of numerous works, in particular [20, 56, 62, 77]. Among the extensions of the present work that require further research, we can mention: (i) models of materials defined by a bipotential (not necessarily stable); (ii) large displacement modeling.

## Data Availability

Not applicable.
